# Design of an inherently-stable water oxidation catalyst

**DOI:** 10.1038/s41467-018-07281-z

**Published:** 2018-11-20

**Authors:** Biswarup Chakraborty, Gal Gan-Or, Manoj Raula, Eyal Gadot, Ira A. Weinstock

**Affiliations:** Department of Chemistry and the Ilse Katz Institute for Nanoscale Science & Technology, Ben-Gurion University of the Negev, Beer Sheva, 84105 Israel

## Abstract

While molecular water-oxidation catalysts are remarkably rapid, oxidative and hydrolytic processes in water can convert their active transition metals to colloidal metal oxides or hydroxides that, while quite reactive, are insoluble or susceptible to precipitation. In response, we propose using oxidatively-inert ligands to harness the metal oxides themselves. This approach is demonstrated by covalently attaching entirely inorganic oxo-donor ligands (polyoxometalates) to 3-nm hematite cores, giving soluble anionic structures, highly resistant to aggregation, yet thermodynamically stable to oxidation and hydrolysis. Using orthoperiodate (at pH 8), and no added photosensitizers, the hematite-core complex catalyzes visible-light driven water oxidation for seven days (7600 turnovers) with no decrease in activity, far exceeding the documented lifetimes of molecular catalysts under turnover conditions in water. As such, a fundamental limitation of molecular complexes is entirely bypassed by using coordination chemistry to harness a transition-metal oxide as the reactive center of an inherently stable, homogeneous water-oxidation catalyst.

## Abstract (web summary)

A current challenge in the development of molecular water oxidation catalysts is to overcome their inherent susceptibilities to oxidative or hydrolytic degradation under turnover conditions in water. Here, the authors design an inherently-stable water oxidation catalyst using oxidatively-inert ligands to harness a reactive metal oxide.

## Introduction

A current challenge in the development of molecular water-oxidation catalysts^1^ is to overcome their inherent susceptibilities to oxidative or hydrolytic degradation under turnover conditions in water^2,3^. Advances in the past decade have led to remarkably fast rates of O_2_ formation^4–6^, along with the parallel development of more stable catalysts^7^, better able to withstand strongly oxidizing conditions in water. Nevertheless, organic ligands are susceptible to oxidation^1,2,8–11^, which, combined with hydrolysis of their active transition-metal centers, often results in the formation of colloidal metal oxides. Entirely inorganic polyoxometalate (POM)-based water-oxidation catalysts^12,13^, by contrast, are thermodynamically stable to oxidation^12–15^, can utilize earth-abundant metals^5,14–17^, and feature remarkably rapid rates^5^. At the same time, molecular POM catalysts are stable to hydrolysis only under specific, albeit well-defined and by now, well-understood conditions, concerning variables such as pH and the nature and concentration of buffer^17–19^. Fundamentally, however, their catalytically active transition metals are in equilibrium with trace concentrations in the aqueous solvent^20^, which can lead to hydrolysis under non-optimal conditions^18^. Hence, as advances in ligand design have led to impressively rapid rates and longer catalyst lifetimes under turnover conditions, catalyst stability nevertheless remains an ongoing topic of discussion and experiment^1,10,20^.

One reason for this heightened level of concern is that colloidal metal oxides can be extremely active^21–23^, such that considerable efforts are required to prove that the molecular catalyst, and not its products of decomposition in water, are the kinetically competent species in catalytic water oxidation. Given this situation, a compelling solution to catalyst stability might be to embrace the colloidal metal-oxide nanocrystals (NCs) themselves as thermodynamically stable water-oxidation catalysts. Apart from a few exceptions^23^, however, colloidal metal oxides are either insoluble at most pH values in water, or rapidly aggregate and precipitate from water under turnover conditions. And, despite impressive advances in solvothermal syntheses of metal-oxide NCs^24^, the requisite organic stabilizing ligands are not only susceptible to oxidation, but usually limit solubility in water and block access to the metal-oxide surface.

In this context, we recently discovered that heteropolytungstate cluster anions (POMs) could serve as covalently attached oxo-donor ligands for 6-nm anatase-TiO_2_ NCs^25^, giving water-soluble POM-complexed nanostructures. We now deploy Fe(III)-substituted [α-P^V^W^VI^_11_O_39_]^7–^ cluster anions as oxo-donor ligands for hematite (α-Fe_2_O_3_) cores, giving water-soluble catalysts, **1**, uniquely positioned between molecular iron-oxide clusters^26^ and colloidal hematite^27,28^. The new catalyst features 3-nm hematite (α-Fe_2_O_3_) cores comprised of ca. 300 Fe atoms, sufficiently large to possess the visible-light semiconductor properties of hematite. Unlike colloidal hematite, however, covalent coordination of each α-Fe_2_O_3_ core by ca. 15 cluster anions renders the POM-complexed structures stable to aggregation in water, giving optically transparent solutions at pH values of 2.5 to 8. Moreover, fitted with entirely inorganic tungsten(VI)-oxide ligands, and formed in water at 220 °C, **1** is thermodynamically stable to both oxidative degradation and hydrolysis. As an inherently stable visible-light activated water-oxidation catalyst, **1** is capable of continuous operation for 7 days under turnover conditions (corresponding to 7600 turnovers) with no decrease in activity.

## Results

### Inorganic coordination complexes of hematite

**Fig. 1:**
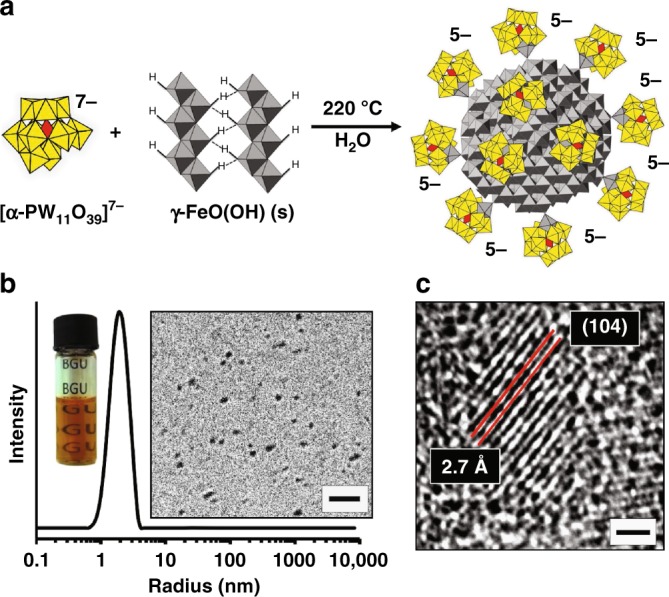
Synthesis of the cluster-anion ligated hematite-core complex, **1**. **a** Synthesis of **1** by reaction of partially amorphous γ-FeO(OH) with α-PW_11_O_39_^7–^ at 220 °C at pH 6.5 in water. An illustration of **1** is shown at the right; gray: Fe(III)-centered polyhedra; yellow: W(VI)-centered polyhedra; red: P(V)-centered tetrahedra. **b** Number-weighted dynamic light scattering of a clear-orange solution of **1** indicated particles with an average hydrodynamic radius of 1.9 nm. The left inset is a photograph of the clear-orange solution; the right inset is a cryo-TEM image of freely soluble particles of **1** in rapidly vitrified water (scale bar: 10 nm). **c** HRTEM image of the hematite core of **1** with fringes corresponding to (104) planes (scale bar: 1 nm)

Complex **1** is prepared by converting micron-sized particles of γ-FeO(OH) to α-Fe_2_O_3_ (at 220 °C) in the presence of [α-PW_11_O_39_]^7–^, after which, numerous Fe(III)-substituted anions, [α-PW_11_O_39_Fe^III^]^4–^–O^–^, remain bound via bridging oxo linkages to 3-nm hematite cores (Fig. 1a and Supplementary Figures 1-7). The reaction gives an orange, optically transparent pH-7 solution of **1** (Fig. 1b, left inset). Dynamic light scattering (DLS) of the clear-orange solution reveals a number-weighted hydrodynamic radius of 1.9 nm (Fig. 1b). Cryogenic-TEM images^29^ of the same (vitrified) solution (right inset to Fig. 1b) reveal numerous freely diffusing particles with an average size of 2.8–3.0 nm, too small for resolution of their surface structures in cryo-TEM images (additional images in Supplementary Figure 8). (This limitation is consistent with extensive studies of POMs on gold nanoparticles, in which even well-ordered POM monolayers are not discernible on gold cores smaller than ca. 5 nm^29^.)

The cores of **1** are single NCs of hematite (α-Fe_2_O_3_). High-resolution TEM images (Fig. 1c) reveal an inter-planar spacing of 2.69 Å, in agreement with the (104) crystal planes of hematite^30^. Indexing of the well-defined rings found in electron diffraction of dry samples precisely matches hematite (Supplementary Figure 9a)^31^, and the related dark-field images reveal individual 2.8 ± 0.5 nm ( ± denotes s.d.) diameter NCs (Supplementary Figure 10). The hematite structure was further confirmed by powder X-ray diffraction (XRD)^30^, for which the Debye–Scherrer equation^32^ gave a crystallite size of 3.5 ± 0.5 nm (Supplementary Figures 9b and 11).

### Isolation and stability to aggregation

To isolate pure samples of **1**, a clear-orange solution (inset to Fig. 1b) was made 2 M in NaCl. This gave a cloudy solution of salted-out **1**, an orange-red solid isolated by centrifugation. Re-dissolution of **1** in pure water (10 mL) returned clear-orange solutions, even after multiple cycles of NaCl addition, centrifugation, and re-dissolution (Supplementary Figure 2). This solubility and remarkable stability to aggregation differs dramatically from the properties of electrostatically stabilized colloids. Behaving more like a molecular macroanion, the hydrated Na^+^ salt of **1** can be stored indefinitely, and then readily dissolved in water. These findings provided the first indication that, as shown in Fig. 1a, heteropolytungstate cluster anions are covalently bound to the hematite cores.

### Cluster-anion ligands coordinated to hematite cores of **1**

**Fig. 2:**
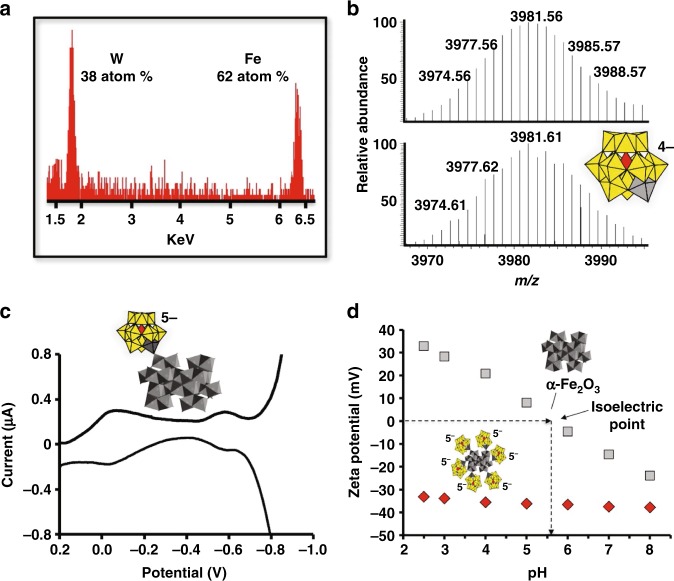
Characterization of cluster-anion ligands on the hematite cores of **1. a** EDX spectrum of **1**. **b** Top: ESI-MS spectrum; bottom: a simulated spectrum, showing a precise match with the Keggin ion [(TBA)_5_{PW_11_O_39_Fe^III^(H_2_O)_2_}]^+^ (see Supplementary Figure 14). Inset: the Keggin anion, with the Fe(III)-centered polyhedron in gray. (In water, the outward-facing coordination site on Fe(III) is occupied by H_2_O or OH^–^, or by a *μ*_2_–O^2–^ linkage to Fe(III) in a second cluster anion.). **c** Differential pulse voltammogram (DPV) of **1** in 100 mM LiClO_4_ and acetate buffer (pH 3.3). Two reversible redox waves (at −50 mV and −590 mV) correspond to the Fe(III/II) redox couple, and to 1-e^–^ reduction of W(VI) in [α-PW_11_O_39_Fe]^4–^ on the surface of the hematite cores. The second (more negative) redox couple of the W(VI) atoms is not well resolved due to overlap with a large cathodic current at −700 mV (possibly due to H_2_ formation, and/or to reduction of Fe(III) in the hematite cores). (See Supplementary Figure 15b for comparison to [(α-PW_11_O_39_Fe^III^)_2_O]^10–^ (**2.**)) **d** The surface zeta potentials (*ζ*, in units of mV) of colloidal α-Fe_2_O_3_ (suspension; gray squares) are positive at low pH values and negative at pH values above the isoelectric point near pH 5.5, as indicated by the dotted lines. By contrast, **1** (red diamonds) is soluble and negatively charged from pH 2.5 to 8. (Atomic connectivity of the cluster-anion ligands to the hematite surface is discussed below.)

Energy-dispersive X-ray spectroscopic (EDX) analysis of **1** (Fig. 2a and Supplementary Figure 12) revealed an atomic composition of 38 ± 5% W and 62 ± 5% Fe. Because the cores are crystalline α-Fe_2_O_3_, the tungsten atoms must reside at the hematite surface. The tungsten-based surface structures were characterized by electrospray ionization mass spectroscopy (ESI-MS). Mild HCl etching of **1** at pH 2 (6 h at 70 °C), gave a cloudy orange solution (Supplementary Figure 13). After removing an insoluble iron oxide by centrifugation, a pale-yellow powder precipitated upon addition of tetra-*n-*butylammonium (TBA) salt. ESI-MS spectra of this solid revealed two dominant sets of signals, centered at *m*/*z* = 3981.56 and 2232.57 amu, with isotope-distribution patterns matching the +1 ion, [(TBA)_5_{PW_11_O_39_Fe(H_2_O)_2_}]^+^ (Fig. 2b) and the 2+ ion, [(TBA)_7_{PW_11_O_39_Fe(OH)(H_2_O)}]^2+^, respectively (Supplementary Figure 14). Both ions are consistent with HCl cleavage of [α-PW_11_O_39_Fe] moieties from the surfaces of the hematite cores.

Differential pulse voltammetry (DPV)^25^ performed after adding LiClO_4_ (0.1 M) to aqueous solutions of **1** (Fig. 2c), provided further support for the presence of [α-PW_11_O_39_Fe] anions at the hematite surface. While conceptually analogous to electrochemical studies of ferrocene-covered SiO_2_ nanoparticles^33^, to our knowledge, the observation of reversible redox chemistries of ligands bound to metal-oxide NCs in solution has few precedents in the literature^25^. It was made possible here by the abundance of cluster anions on the hematite surface, in combination with the water solubility imparted by their negative charges and numerous alkali-metal counter-cations. After adding LiClO_4_ (0.1 M), differential pulse voltammetry (DPV) revealed reversible redox processes consistent with the presence of the cluster anions identified by ESI-MS (DPV data for the independently prepared molecular cluster anions are provided in Supplementary Figure 15b).

Surface coverage by the POM anions, based on a reasonable footprint of 1.9 nm^2^, places 15 POMs on the surface of an (idealized) spherical 3-nm-diameter hematite core (Supplementary Table 1). Such a structure would contain 165 W atoms and 279 Fe atoms, giving a relative atomic composition of 37% W and 63% Fe, nicely matching the %-atom values observed by EDX (Fig. 2a). The W to Fe ratio also gives ca. 18–20 Fe atoms per POM ligand.

Not only is **1** remarkably stable to aggregation, it is soluble at pH values of 2.5–8. Over this wide pH range, its largely negative zeta potential values (*ζ*, −35 to −40 mV) remain pH-invariant (Fig. 2d). By contrast, colloidal α-Fe_2_O_3_ precipitates at its isoelectric point (pH 5.5), at which *ζ* = 0 mV^34^. These findings represent three independent lines of evidence for strong coordination of the POM ligands to the α-Fe_2_O_3_ cores.

Given these inert linkages, metathesis of Na^+^ counter-cations (of the POMs) by *n*-R_4_N^+^ (R = hexyl or octyl) was used to render **1** organic-solvent soluble. This cation-exchange procedure—typical of POM salts—gave optically transparent MeCN or MeOH solutions with no loss of POM ligands (Supplementary Figure 16), further evidence for their covalent attachment to the hematite cores.

It is extraordinarily difficult to precisely determine the atomic connectivities of molecules bound to colloidal NCs. However, the presence of a *μ*_2_-oxo linkage between Fe(III) ions in the known oxo-bridged dimer, [(α-PW_11_O_39_Fe)_2_–*μ*_2_-O]^10–^ (**2**)^35^, suggested that a similar linkage might bind oxo-donating [α-PW_11_O_39_Fe^III^]^4–^–O^–^ ligands to Fe(III) atoms at the α-Fe_2_O_3_ surface. X-ray photoelectron spectroscopy (XPS) data for **1** revealed a 2.1 eV difference between W4f_7/2_ and W4f_5/2_ binding energies (Supplementary Figure 17). Notably, these W4f peaks are comparable with those of **2** (Supplementary Figures 18 and 19), consistent with the presence of Keggin-anion derived ligands connected to the surface of **1** via *μ*_2_-oxo linkages.

**Fig. 3:**
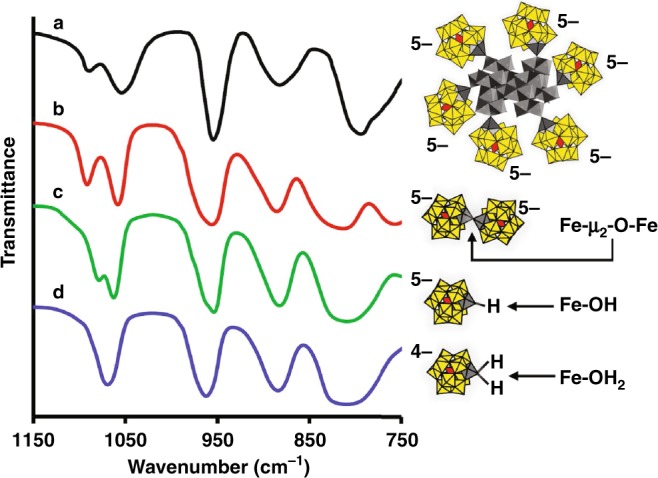
FTIR spectral evidence for Fe–*μ*_2_-O–Fe linkages to α-[PW_11_O_39_Fe]–O^–^ ligands in **1**. **a** and **b** FTIR spectra, respectively, of **1** (baseline corrected—see Supplementary Figure 20a) and the *μ*_2_-oxo-bridged dimer, [(α-PW_11_O_39_Fe)_2_–*μ*_2_-O]^10–^ (**2**). (The lowest-energy band in **a** is distorted by overlap with bands from α-Fe_2_O_3_). The IR-active PO_4_ modes in **a** and **b** match one another, but differ substantially from those of monomeric [α-PW_11_O_39_Fe^III^L]^n–^, L = hydroxo or aquo (**c** and **d**, respectively)

This was investigated in more detail by FTIR spectroscopy (Fig. 3). The IR-allowed vibrational modes of the central PO_4_ moieties within Keggin-derived structures are highly sensitive to changes in symmetry of the cluster anion. Removal of a single W(VI) ion from the plenary-Keggin anion, [α-PW_12_O_40_]^3–^, reduces the local symmetry of the central PO_4_ moiety from *T*_*d*_ to *C*_*3v*_. As a result, rather than a single IR-active mode at 1080 cm^–1^, two IR-active PO_4_ modes are observed for mono-lacunary [α-PW_11_O_39_]^7–^. And, after complexing a transition-metal cation, the difference in energy between the two IR-active modes depends on cation size, and the degree to which it fits within the lacunary anion’s pentacoordinate binding site^36^. As the ion is pulled farther out from the binding pocket, the local symmetry around the central PO_4_ moiety deviates from *Td*, leading to a larger difference in energy between the two IR-active PO_4_ bands. Hence, splitting of the PO_4_ mode can be used to identify the ligand environments of substituted transition-metal cations.

As such, the FTIR spectrum of **1** (Fig. 3a; after baseline correction as shown in Supplementary Figure 20a), was compared with those of the μ-oxo-bridged dimer, [(α-PW_11_O_39_Fe^III^)_2_–μ_*2*_-O]^10–^ (**2**; Fig. 3b), and of monomeric POMs with aqua and hydroxo ligands, Fe^III^(OH_2_) and Fe^III^(OH) (Fig. 3, c and d).

The precise match between the PO_4_ bands^36^ of the POM ligands on **1** (1090 and 1051 cm^−1^; Fig. 3a) with the corresponding PO_4_ bands of **2** (1090 and 1050 cm^−1^; Fig. 3b), is consistent with *μ*_2_-oxo linkages between the POM ligands and the hematite cores. Combined with the EDX data in Fig. 2a, this reasonable atomic connectivity suggests structures of the form, {[α-PW_11_O_39_Fe]-*μ*_2_-O}_~15_-(α-Fe_2_O_3_)_~150_, in which ca. 15 POMs are bound to cores comprised of ca. 150 Fe_2_O_3_ units.

This covalent attachment of POM cluster anions is fundamentally distinct from the use of POMs to electrostatically stabilize colloidal metal-oxide NCs. Notably, in a series of seminal papers, Murray^37^, Milleron and Helms^38^, and Talapin^39^ used NOBF_4_, Me_3_OBF_4_, and Ph_3_CBF_4_, respectively, to replace organic protecting ligands on metal-oxide NCs by electrostatically associated BF_4_^–^ anions in polar organic solvents^40^. Milleron^41,42^ and Talapin^39^ then replaced the weakly bound BF_4_^–^ anions by hexaniobate and other POMs, respectively, giving clear solutions of NCs electrostatically stabilized by the POM anions. Having removed the more tightly bound organic ligands, Milleron used hexaniobate-stabilized Sn-doped In_2_O_3_ (ITO) NCs as building blocks for tunable nanocrystal-in-glass composites^41,42^, while Talapin showed that films prepared by depositing POM-stabilized Fe_2_O_3_ on ITO electrodes were more effective electrocatalysts for water oxidation than analogous ones prepared from organic-ligand protected NCs^39^.

In the present work, the covalent attachment of POM ligands to the hematite cores of **1** gives substitutionally inert structures that occupy a unique position at the interface between molecular iron-oxide clusters and electrostatically stabilized colloidal iron-oxide NCs. We now show that, when used as a soluble photocatalyst for visible-light driven water oxidation, **1** is inherently stable under turnover conditions in water.

### Catalytic water oxidation

The activity of **1** as a visible-light water-oxidation catalyst was explored by irradiating pH-8 solutions of **1** with visible light (150 W Xe lamp, cutoff *λ* ≥ 420 nm, Supplementary Figures 21 and 22). Four oxidants were evaluated: Ce(IV), Ag^+^, S_2_O_8_^2–^, and periodiate (I^VII^O_4_^–^). Ce(IV) was not suitable as it is only stable at pH values below 1, at which hematite itself dissolves. Although Ag^+^ is soluble at neutral pH values, these large cations form strong ion pairs with the negatively charged POM ligands, leading to precipitation at desired Ag^+^ concentrations. By contrast, **1** is soluble in 20 mM solutions of the anionic oxidants. For S_2_O_8_^2–^ and IO_4_^–^, 1025 and 6330 μmol O_2_ per gram of α-Fe_2_O_3_ in **1**, respectively, were produced in 8-h reactions (Table 1, entries 1 and 2; Supplementary Figure 23).

**Table 1:** Visible-light driven water oxidation by **1**^a^

Entry	Oxidant^b^	Catalyst	Light^c^	Solvent	O_2_ (μmol g^−1^)^d^
1	S_2_O_8_^2–^	**1**	Visible	H_2_O	1025
2	IO_4_^–^	**1**	Visible	H_2_O	6300
3	None	**1**	Visible	H_2_O	0
4	IO_4_^–^	None	Visible	H_2_O	0
5	IO_4_^–^	**1**	Dark	H_2_O	0
6	IO_4_^–^	{[α-PW_11_O_39_Fe]_2_O}^10–^ (**2**)^e^	Dark	H_2_O	60 ± 20
7	IO_4_^–^	{[α-PW_11_O_39_Fe]_2_O}^10–^ (**2**)^e^	Visible	H_2_O	60 ± 20
8	IO_4_^–^	Fe^3+ f^	Visible	H_2_O	0
9	IO_4_^–^	**1** ^g^	Visible	Dry MeCN	0
10	IO_4_^–^	**1** ^g^	Visible	1:1 H_2_O:MeCN	1640

^a^All reactions were carried out at pH 8 for 8 h (40 ± 1 °C)

^b^Oxidant concentrations were 20 mM

^c^Light source was a 150 W Xe lamp with a *λ* ≥ 420-nm cutoff filter

^d^Values reported are per gram of the α-Fe_2_O_3_ cores, or of the catalysts listed in column three

^e^These two control experiments (entries 6 and 7) rule out oxygen evolution by reaction of periodate with the Fe(III) atoms complexed within the hematite-bound POM ligands. They were carried out at pH 5 to ensure integrity of the molecular dimer, **2**. In 8 h at pH 5, **1** gave 3200 μmol O_2_ g^−1^

^f^After air oxidation of FeSO_4_ at pH 5 and 8

^g^Carried out using an organic-solvent soluble form of **1**, and R_4_N^+^IO_4_^–^ (R = *n*-butyl)

Although written as IO_4_^–^, a recent report shows^43^ that its dominant form in water is actually orthoperiodate, H_5_IO_6_, which behaves as a polyprotic acid, with pKa_1_, pKa_2_, and pKa_3_ values of ca. 1, 7.5, and 11, respectively. For simplicity, entries in Table 1 refer to added periodate (IO_4_^–^).

The greater reactivity of IO_4_^–^ relative to S_2_O_8_^2–^ (Table 1) is not unique to visible-light driven reactions of **1**. Notably, IO_4_^–^ is also more effective than S_2_O_8_^2–^ in trapping photoexcited electrons from visible-light irradiated WO_3_^44^, Ru(bpy)_3_^2+^, and Fe(bpy)_3_^2+^^45^. Specifically, visible-light driven electron transfer (ET) from Ru(bpy)_3_^2+^ to IO_4_^–^ is twice as fast as the corresponding reduction of S_2_O_8_^2–^, and for Fe(bpy)_3_^2+^, visible-light driven ET to IO_4_^–^ is two orders of magnitude faster than ET to S_2_O_8_^2–^^45^.

### Support for periodate acting as a two-electron acceptor

For the reaction in entry 2, two equivalents of IO_4_^–^ are reduced to IO_3_^–^ for each equivalent of evolved O_2_ (Supplementary Figure 24), consistent with IO_4_^–^ acting as a 2-e^–^ oxidant. Moreover, all three components (IO_4_^–^, **1**, and visible light) are required for O_2_ formation (entries 3–5), and the quantum yield (3.9%) is typical of water oxidation by hematite nanocrystals (Supplementary Table 2). At the same time, when using IO_4_^–^^11^, decomposition or oxo-transfer reactions^46^, that could generate O_2_ without removing four electrons from water, must be ruled out^47,48^. This required numerous control experiments (see Supplementary Figure 25 and Table 3), necessitated by the fact that the oxide ligands of periodate equilibrate rapidly with water, precluding the use of ^18^O-labeling to definitively prove that the oxygen atoms in evolved O_2_ originate from H_2_O^49^. The results of the control experiments performed are fully consistent with the clean two-electron reduction of IO_4_^–^ to IO_3_^–^.

In brief, photochemical decomposition of IO_4_^–^ (capable of generating O_2_) occurs only under UV irradiation^50^. When irradiated with visible light (*λ* ≥ 420 nm), no decomposition of IO_4_^–^ was observed, and no O_2_ was detected (Table 1; entry 4). Secondly, oxo-transfer mechanisms (noted above) invariably involve thermal (dark) reactions of molecular Fe, Ru, and Ir complexes^46–48,51^. By contrast, dark reactions of IO_4_^–^ with **1** gave no O_2_ (entry 5), while the dark reaction of IO_4_^–^ with {[α-PW_11_O_39_Fe]_2_O}^10–^ (**2**) gave only traces of O_2_ (entry 6). The same reaction in visible light (entry 7) also gave traces of O_2_, probably due to the dark reaction. These findings definitively rule out catalysis by the [α-PW_11_O_39_Fe^III^]^4–^–O^–^ ligands bound via oxo linkages to the α-Fe_2_O_3_ surface, including via oxo transfer from periodate to the POM-complexed Fe(III) atoms. In addition, no O_2_ is produced in dark reactions of IO_4_^–^ with γ-Fe(O)OH^52^ or α-Fe_2_O_3_^28^, and no O_2_ was detected when IO_4_^–^ was reacted under visible light with partially hydrolyzed Fe^3+^ (entry 8).

To confirm that ET to IO_4_^–^ does not generate O_2_, periodate was stoichometrically reduced to IO_3_^–^ by two equivalents of the 1-e^–^ donor, [α-AlV^IV^W_11_O_40_]^7–^^53^. No O_2_ was detected (Supplementary Figure 26a). To model the trapping of photoexcited electrons from **1**, [Ru(bpy)_3_]^2+^ was reacted with IO_4_^–^. While no reaction occurred in the dark, visible-light (*λ* ≥ 420 nm) promoted the 2-e^–^ reduction of IO_4_^–^ to IO_3_^–^. Again, no O_2_ was detected (Supplementary Figure 26b). Finally, direct evidence that water is required for O_2_ formation was obtained by reacting organic-solvent-soluble forms of **1** and IO_4_^–^ in dry and wet MeCN (entries 9 and 10; see Supplementary Figure 27)^51^. Although less definitive than isotope labeling, the results show that H_2_O is necessary for O_2_ formation, consistent with H_2_O as the source of O_2_ in pure water.

### Rate optimization and stability under turnover conditions

**Fig. 4:**
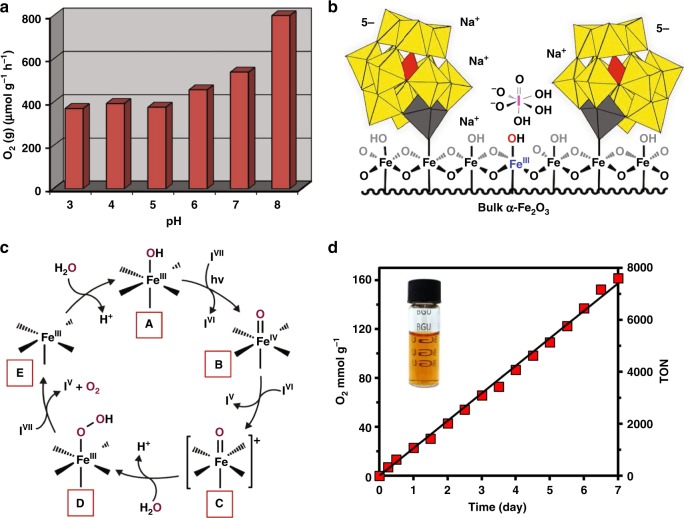
Visible-light driven water oxidation by **1**. **a** Rate of O_2_ formation (per gram of α-Fe_2_O_3_ in **1**, per hour), for 2.4 μM **1** and 20 mM NaIO_4_, as a function of pH. **b** Scale model showing the relative sizes and plausible locations of cluster-anion ligands, [α-PW_11_O_39_Fe]–O^–^, Na^+^ counter-cations, and orthoperiodate ([H_3_I^VII^O_6_]^2–^) at a surface of the α-Fe_2_O_3_ core of **1**. The Fe(III) atoms at the hematite surface are six coordinate, and at pH 8, are terminated by hydroxide ligands. **c** A plausible catalytic cycle for photochemical water oxidation, highlighting proposed elementary steps, with the active site comprised of a single Fe^III^ atom at the surface of the Fe_2_O_3_ core (highlighted by blue text in panel b). See text for details. **d** Dioxygen produced by **1** (5.4 μM) as a function of time during 7 days of reaction at pH 8. Oxygen production is plotted in mmol O_2_ per g of α-Fe_2_O_3_ in **1** (as routinely done for colloidal metal-oxide catalysts), and the turnover number (TON) is defined as mol O_2_ per mol **1**, as is typical for molecular catalysis. The initial concentration of NaIO_4_ was 20 mM. During the course of the 1-week reaction, the solution was periodically charged with additional NaIO_4_, and separated from accumulated iodate (IO_3_^–^) (see the Methods section for details). The starting pH value and those after 7 days, were 8.0 and 8.2, respectively; the buffering was provided by orthoperiodate, for which p*K*a_2_ and p*K*a_3_ are ca. 7.5 and 11, respectively^44^

Unlike most polyoxometalate-based water-oxidation catalysts, **1** is stable in water over a wide range of pH values, from 2.5 to 8. And, in contrast to colloidal hematite that precipitates from solution at its isoelectric point (i.e., at pH 5.5), **1** is soluble over this entire pH range (see Fig. 2d). Hence, unlike most purely molecular or traditional colloidal catalysts, the activity of **1** can be investigated over a wide range of pH values. The results (Fig. 4a) reveal an approximate doubling of rate from pH values of 5 to 8. At pH 8, the rate of O_2_ formation is 800 ± 50 µmol g^−1^ h^−1^ (at *λ* ≥ 420 nm using a 150 W Xe lamp), somewhat exceeding the fastest reported rates for visible-light driven water oxidation by colloidal α-Fe_2_O_3_ (see Supplementary Table 4 for reported values and related light sources).

The change in pH from 5 to 8 spans the isoelectric point of hematite (near pH 5.5; Fig. 2d). As such, the increase in rate may correlate with deprotonation of water molecules bound to the hematite surface, to give more reactive (negatively charged) hydroxide ligands, in combination with the more favorable, pH-dependent Gibbs free energy for water oxidation itself. For periodate to trap photoexcited electrons, it must diffuse to within a close proximity to the α-Fe_2_O_3_ surface. The cluster-anion ligated surface of **1**, including [H_3_I^VII^O_6_]^2–^ (orthoperiodate—the dominant periodate species present at pH 8—drawn to scale), and Na^+^ counter-cations (without their hydration shells), is illustrated in Fig. 4b, with iron atoms at the hematite surface shown with the terminal hydroxide ligands that dominate at pH 8. Orthoperiodate is smaller than hydrated Na^+^ cations;^54^ the radius of six coordinate I(VII) is 0.67 Å, compared to 1.16 Å for Na(I) in the aquo complexes, [Na(H_2_O)_6_]^+^, likely located between the negatively charged POM ligands. As such, [H_3_I^VII^O_6_]^2–^ anions, and/or hydrogen-bonded ion pairs such as [(H_2_O)_5_NaOH_2_–O_2_I(O)(OH)_3_]^–^, should easily approach Fe^III^–OH moieties at the hematite surface.

The rate of visible-light driven water oxidation by **1** is enhanced by the relatively small size of the hematite cores, which is critically important^28^ due to the short, 2–4 nm, hole-diffusion length^55^ of α-Fe_2_O_3_. This leads to a quantum yield of 3.9%, closely matching values reported for optimized reactions of similarly sized colloidal hematite^28,56^.

A preliminary schematic of reasonable mechanistic steps is provided in Fig. 4c. Trapping of an excited electron by [H_3_I^VII^O_6_]^2–^ (the dominant form of periodate at pH 8)^43^ would result in oxidation of a surface Fe^III^–OH moiety to Fe^IV^=O (**A** and **B**, respectively, in Fig. 4c), a species recently confirmed by operando infra-red spectroscopy^27^ to be an intermediate in photoelectrochemical water oxidation on hematite films^57,58^. The subsequent one-electron oxidation of Fe^IV^=O by the reactive I(VI) radical (in a dark reaction) would give a species abbreviated as [Fe=O]^+^ (**C**), whose precise electronic structure is unknown^27,59^. Computational results argue that distances between adjacent Fe atoms at the hematite surface are too large to give stable peroxide-bridged di-iron intermediates, Fe^III^-OO-Fe^III^^60^. At the same time, the first and third-order dependence of hole formation reported by Durrant^61^, and the first- and second-order kinetics and operando observations of electrochemical water oxidation by Chen and Song^62^, support O–O formation both at a single Fe atom, and at larger hole densities and/or highly basic pH values, via coupling between adjacent surface-trapped holes. For solutions of **1** at pH 8, O–O formation is more likely to occur at a single Fe atom, giving the iron(III)-hydroperoxide intermediate, Fe^III^-OOH (**D**). Dioxygen is then generated by two-electron oxidation of Fe(III)-bound hydroperoxide ligand, resulting in **E**, which reacts rapidly with water to give **A**.

Throughout four consecutive 8-h reactions (a total of approximately 1400 turnovers per equivalent of **1** over 32 h), the turnover frequency (TOF) remained constant (44 ± 1 h^–1^) and the solutions remained optically transparent. After each 8-h reaction, the catalyst was quantitatively isolated by salting out with NaCl (2 M) and centrifugation, followed by re-dissolution in fresh water (Supplementary Figure 28a). Notably, this had no effect on its activity. The same remarkable retention of activity was observed in a single 7-day (1-week) reaction (7600 turnovers; Fig. 4d). No decrease in activity was observed. The TOF remained constant at 44 ± 1 h^–1^, the solution remained optically transparent (inset to Fig. 4d), and no aggregation was observed by DLS (Supplementary Figure 29).

The TON of 7600 is much larger than that reported for colloidal hematite. For example, under identical conditions, the initial rate of O_2_ formation by 5-nm colloidal α-Fe_2_O_3_ (no POM ligands)^56^ was ca. 320 µmol g^−1^ h^−1^, but decreased dramatically within a few hours due to aggregation processes typical of colloidal metal oxides in water (Supplementary Figure 28b). Moreover, the present TON is more than seven times that reported for the most stable, water-soluble Fe-based catalysts^11^ which, although much faster than **1** (with respect to TOF), become inactive within a few hours under turnover conditions in water. And, while acknowledging that **1** contains ca. 300 Fe atoms, many more than typically found in traditional molecular catalysts, its relatively small (ca. 20 Å) α-Fe_2_O_3_ center is nevertheless large enough to retain the photochemical properties of bulk hematite. It is from this perspective that **1** can be viewed as a soluble complex of a reactive metal-oxide core.

## Discussion

Oxidatively inert heteropolytungstate cluster anions serve as covalently coordinated oxo-donor ligands for 3-nm hematite (α-Fe_2_O_3_) cores, giving anionic complexes soluble in water over a wide range of pH values (from 2.5 to 8), and that catalyze visible-light driven water oxidation with no need for added photosensitizers. Like molecular macroanions, **1** is highly resistant to aggregation processes that typically lead to the precipitation of electrostatically stabilized hematite and other colloidal metal oxides. And, formed by reaction with entirely inorganic tungsten-oxide-based ligands at 220 °C in water, **1** is inherently (thermodynamically) stable to the oxidative and hydrolytic processes that can limit the active lifetimes of molecular water-oxidation catalysts. As such, **1** can continuously catalyze water oxidation for 7 days (1 week) with no detectable decrease in activity. Moreover, the method used to prepare **1** is not limited to iron oxide, but can be modified according to the pH-controlled aqueous speciation chemistries of numerous other transition-metal ions. As such, the covalent coordination of oxidatively inert polyoxometalate ligands to metal-oxide nanocrystal cores represents a conceptually new and general approach to the design of inherently stable water-oxidation catalysts.

## Methods

### Preparation of **1**

Iron(II) sulfate (FeSO_4_·7H_2_O, 16.7 mg, 0.06 mmol) was dissolved into 8 mL of deionized water, and stirred for 1 hour, giving a pale-yellow solution. Freshly prepared 0.2 N KOH (2.2 equiv, 700 µL) was then added dropwise with vigorous stirring, resulting in the formation of an orange-red pH 7.0 suspension of partially amorphous γ-FeO(OH) (s). After 20 h of vigorous stirring at room temperature, 750 μL of 40 mM Na_7_[α-PW_11_O_39_] (0.03 mmol) was added dropwise, and 550 μL of deionized water was added to reach a final volume of 10 mL, and stirred for 1 h at room temperature, giving an orange pH 6.7 suspension. The suspension (6 mM Fe(III) and 3 mM Na_7_[α-PW_11_O_39_]), was then heated for 24 h at 220 °C in a 23-mL Teflon-lined 316 stainless-steel reaction vessel, to give a clear orange-red, pH-6.5 solution of **1**. Details of isolation and purification are provided as Supplementary Materials and Methods.

### Visible-light driven water oxidation

Photochemical O_2_ production was carried out in a gas-tight quartz cuvette connected to an upper glass bulb with a headspace volume of 16 mL. Carefully weighed samples of **1** were dissolved in 2.7 mL of water along with sodium periodate (NaIO_4_; 12.8 mg in 0.1 mL of water) to give a final NaIO_4_ concentration of 20 mM. The addition of NaIO_4_ led to a drop in pH from 6.5 to 5, and either 0.2 N KOH or 0.2 N HCl solutions were used to adjust the pH to desired values (see Fig. 4a). The headspace within the cuvette was evacuated and refilled with pure Ar (**g**) four times to remove most of the air, after which the solution was purged with Ar (**g**) for 30 min to remove dissolved oxygen. The solution was then irradiated with a 150 W Xe lamp (USHIO Inc. Japan), after inserting a 420-nm long-pass filter between the light source and the cuvette, which was held at a distance of 12 cm from the light source. The illumination area was 2 cm^2^ and the average light intensity was 565 mW cm^−2^ at *λ* = 450 nm measured using a 1928-C Optical Power Energy Meter (Newport Corp.) equipped with a model 919P-250-35 Thermopile sensor (UV-calibrated silicon detector). The headspace gas (0.5-mL volume portions) was injected every 2 hours into a gas chromatograph (Focus GC, Thermo Scientific) operating at isothermal conditions (40 °C) using a ShinCarbon ST micropacked column (0.53-mm diameter, 2-m length), and equipped with a thermal conductivity detector (TCD) and Ar as carrier gas. Moles of O_2_ per gram reported for **1** were calculated based on the mass of its α-Fe_2_O_3_ cores, which comprised ca. 50% of the total mass of **1**.

### Water oxidation for 7 days under turnover conditions

A solution of **1** (5.4 μM) and NaIO_4_ (20 mM) in 3 mL of water at pH 8 (adjusted using 0.2 N KOH) was degassed as described above and irradiated with visible light (*λ* > 420 nm) for 1 day, during which, amounts of O_2_ in the headspace were quantified at regular intervals (see Fig. 4d). After 1 day, 6.4 mg of solid NaIO_4_ dissolved in 0.1 mL of water was added to the solution (an additional 10 mM concentration of IO_4_^–^) and the reaction continued for a second day. After day 2, **1** was quantitatively separated from accumulated iodate (IO_3_^–^; ca. 5.5 mM) by making the solution 0.5 M in NaCl, and isolating the salted-out catalyst by centrifugation. (A control experiment later carried out after recharging the supernatant solution with periodate, followed by irradiation, showed no activity (i.e., no O_2_ in 8 h). This demonstrated that possibly unidentified soluble components were not responsible for the catalysis.) The pellet of **1** isolated after day 2 was then dissolved in 3 mL of water containing freshly added periodate (20 mM), adjusted to pH 8, degassed, and irradiated for day 3. (Similar separations of **1** from accumulated IO_3_^–^ were repeated after days 4 and 6.) After days 3 and 5, additional 10 mM concentrations of IO_4_^–^ were added. Turnover numbers were calculated based on the moles of O_2_ produced per mole of **1**.

## Electronic supplementary material

Supplementary Information

Peer Review File

## Data Availability

All data generated or analyzed during this study are included in this published article (and its supplementary information files).
