# Supplementary material for: Design of an inherently-stable water oxidation catalyst
Source: Nat Commun. 2018 Nov 20;9:4896. doi: 10.1038/s41467-018-07281-z (PMC6244296; doi:10.1038/s41467-018-07281-z)
Supplement: Supplementary file 1 — Supplementary Information [file 41467_2018_7281_MOESM1_ESM.pdf]

Supplementary Information for:

Design of an inherently-stable water oxidation catalyst

Authors: Biswarup Chakraborty, Gal Gan-Or, Manoj Raula, Eyal Gadot and Ira A. Weinstock*

Affiliation: Department of Chemistry and the Ilse Katz Institute for Nanoscale Science & Technology, Ben-Gurion University of the Negev, Beer Sheva 84105, Israel.

Corresponding authors:
*Email: iraw@bgu.ac.il

Contents

Supplementary Materials and Methods, including:
General Instrumentation and Analytical Methods.
Isolation and purification of **1**.
Characterization of **1**.
Characterization of the reaction byproduct, $[\alpha\text{-PW}_{11}O_{39}Fe(OH)]^{5-}$.
Preparation of organic-solvent soluble n-R_4N^+ substituted **1**.
Calculation of the quantum efficiency for photochemical O_2 production.
Photochemical reaction in MeCN and in a MeCN-H_2O (1:1, v:v) mixture.
Catalytic water oxidation for three days under turnover conditions.

Supplementary Figure 1: Characterization of partially amorphous γ-FeO(OH) (s) by XRD, FTIR, Raman, and XPS.

Supplementary Figure 2: Step-by-step visual monitoring (photographs) after reactions leading to an optically transparent orange solution of $[\alpha\text{-PW}_{11}O_{39}Fe]^{4-}$-O-$Fe_2O_3$ cores.

Supplementary Figure 3: P-31 NMR quantification of free phosphate, an expected byproduct formed during the reaction of amorphous γ-FeO(OH) (s) with $Na_7[\alpha\text{-PW}_{11}O_{39}]$.

Supplementary Figure 4: Evidence for the byproduct, $[\alpha\text{-PW}_{11}O_{39}Fe(OH)]^{5-}$, obtained by comparing UV-vis spectra of supernatant solutions (from reaction mixtures) with those of independently prepared $[\alpha\text{-PW}_{11}O_{39}Fe(H_2O)]^{4-}$, $[\alpha\text{-PW}_{11}O_{39}Fe(OH)]^{5-}$, $[(\alpha\text{-PW}_{11}O_{39}Fe)_2O)]^{10-}$ and $[\alpha\text{-PW}_{11}O_{39}]^{7-}$.

Supplementary Figure 5: Cyclic voltammetry (CV) and difference pulse voltammetry (DPV) of the reaction mixture after precipitation of **1** by addition 2 M NaCl.

Supplementary Figure 6: Identification of the byproduct, $[\alpha\text{-PW}_{11}O_{39}Fe(OH)]^{5-}$, by ESI-mass spectroscopy.

Supplementary Figure 7: Characterization of the byproduct, $[\alpha\text{-PW}_{11}O_{39}Fe(OH)]^{5-}$, by FTIR spectroscopy.

Supplementary Discussion: Balanced equation for the formation of **1**.

Supplementary Figure 8: Cryo-TEM images of small aggregates of **1**.

Supplementary Figure 9: Characterization of the hematite core by electron diffraction, XRD and HRTEM.

Supplementary Figure 10: Bright-field TEM image of individual cores, selected-area electron diffraction, and a corresponding dark-field TEM image.

Supplementary Figure 11: TEM images of hematite cores of a dried sample and the particle-size distribution.

Supplementary Figure 12: High-angle annular dark field (HAADF) image of **1**, and determination of atom-percentages by small-area EDX spectroscopy.

Supplementary Figure 13: UV-visible spectra of solutions obtained after etching **1** with dilute HCl.

Supplementary Figure 14: ESI mass spectra of a TBA salts of $\{PW_{11}O_{39}Fe\}^+$ isolated after etching **1** with dilute HCl.

Supplementary Figure 15: Differential pulse voltammograms (DPVs) of **1** and of molecular $[(\alpha\text{-PW}_{11}O_{39}Fe)_2O)]^{10-}$.

Supplementary Table 1: Calculation details for determining atom-percentages of W and Fe for **1** (based on EDX data).

Supplementary Figure 16: Preparation and characterization of tetra-alkylammonium cation salts of **1**.

Supplementary Figure 17: High resolution X-ray photoelectron spectra of **1**.

Supplementary Figure 18: High resolution X-ray photoelectron spectra of **2**.

Supplementary Figure 19: Comparison of high resolution W4f scans of **1** and **2**.

Supplementary Figure 20: Characterization of **1** by vibrational spectroscopy.

Supplementary Figure 21: Detection of oxygen by GC during photochemical reaction with **1** in presence of $NaIO_4$.

Supplementary Figure 22: Calibration curves used to quantify the amount of O_2 produced during photochemical reactions.

Supplementary Figure 23: Photochemical water oxidation with **1** in the presence of two different oxidants (persulfate and periodate) and at different pH values.

Supplementary Figure 24: Quantification of sodium periodate after photocatalytic water oxidation by **1**.

Supplementary Table 2: Calculation of the quantum yield for photochemical O_2 production with **1**.

Supplementary Figure 25: ESI-mass spectra of a solution of IO_4^- in CH_3CN and $H_2{}^{16}O$, in the presence of $H_2{}^{18}O$.

Supplementary Table 3: Abundance and relative intensities of molecular ion peaks in ESI mass spectra from combinations of periodate ion (IO_4^-) and $H_2{}^{18}O$ in acetonitrile and water.

Supplementary Figure 26: Chemical oxidation of $[\alpha\text{-AlV}^{IV}W_{11}O_{40}]^{7-}$ to $[\alpha\text{-AlV}^{V}W_{11}O_{40}]^{6-}$ by one-half equivalent $NaIO_4$, and photochemical oxidation $[Ru^{II}(bpy)_3]^{2+}$ by IO_4^-.

Supplementary Figure 27: Visible-light driven water oxidation in water, and in dry and wet MeCN.

Supplementary Table 4: Reported photochemical oxygen production rates for $\alpha\text{-}Fe_2O_3$ NCs.

Supplementary Figure 28: Dioxygen formed as a function of time during four successive reactions and visible-light driven water-oxidation catalysis by **1** compared to catalysis by 5-nm colloidal α Fe_2O_3.

Supplementary Figure 29: DLS data of **1** after long photochemical-reaction times.

Supplementary References

Supplementary Materials and Methods

Materials. Iron(II) sulfate heptahydrate ($FeSO_4 \cdot 7H_2O$, J. T. Backer), iron(III) nitrate nonahydrate ($Fe(NO_3)_3 \cdot xH_2O$, Sigma-Aldrich), NaCl (analytical grade, Frutarom, Israel), deuterium oxide (99.9% D, Cambridge Isotope Laboratories, UK), tetraethylammonium chloride (TEA-Cl, Sigma), tetrabutylammonium bromide (TBA-Br, ACS reagent, ≥ 98%, Sigma-Aldrich), tetrabutylammonium hydrogensulfate (TBA-HSO_4, ≥98%, Sigma-Aldrich), tetrahexylammonium bromide (n-Hex_4N-Br, Fluka), tetraoctylammonium bromide (n-Oct_4N-Br, Sigma), acetonitrile (99.8%, Alfa-Aesar), HCl (analytical grade, Bio Lab, Ltd., Israel) and Sodium periodate ($NaIO_4$, 98%, Sigma) were used as received. Sodium tungstate dehydrate ($Na_2WO_4 \cdot H_2O$, extra pure) was purchased from Merck. $[Ru(bpy)_3]Cl_2 \cdot 6H_2O$ (99.95%) was purchased from Sigma and used as received. Additional reagent-grade salts, acids and diethyl ether for polyoxometalate synthesis and reactions were obtained from commercial sources and used as received. Regenerated-cellulose dialysis membranes (45-mm flat-width tubes; 12−14000 Da MWCO) were purchased from VWR Scientific, treated with sodium bicarbonate (Merck) and ehtylenedimainetetraaceticacid (Sigma) before use to remove glycerin and traces of sulfur compounds, and stored in water at 5°C. Water used for cleaning, synthesis and reactions was of high purity (18.2 MΩ resistivity) from a Millipore Direct-Q water-purification system. $Na_7[\alpha\text{-}PW_{11}O_{39}] \cdot 12H_2O$,[1] was prepared by literature methods and checked by FTIR and ^{31}P NMR spectroscopy, the latter giving clean NMR spectra for the different nuclei involved, with signals at -10.8, ppm (ppm-reference solution is given below). $Na_4[\alpha\text{-}PW_{11}O_{39}Fe(H_2O)] \cdot xH2O$, $Na_{10}[(\alpha\text{-}PW_{11}O_{39}Fe)_2O]$, and $Na_5[\alpha\text{-}PW_{11}O_{39}Fe(OH)]$[2-5] were prepared by literature procedures and TBA-salts of the complexes were prepared as light yellow solid by adding excess TBABr to the aqueous solution of the sodium or potassium salt of the cluster anions, followed by washing two to three times with water. All the complexes were characterized by FTIR spectroscopy. $[\alpha\text{-AlV}^{(IV)}W_{11}O_{40}]^{7-}$ was prepared according to the procedure reported in the literature for $[\alpha\text{-AlV}^{(V)}W_{11}O_{40}]^{6-}$, only that the ozone oxidation step was not carried out.[6] Approx. 5 nm (diameter) $\alpha\text{-}Fe_2O_3$ nanocrystals were prepared following the procedure reported in literature.[7]

General Instrumentation and Analytical Methods. EuTech pH 510 Bench-Top pH meter was used to determine the pH values of the reaction solutions. Optical (UV-vis) spectra were recorded using a HP 8452A spectrophotometer equipped with a diode-array detector (190-1100 nm range). P-31 nuclear magnetic resonance (NMR) spectra were acquired on a Bruker 400 MHz instrument and the chemical-shift values were externally referenced, to 1.0 M H_3PO_4 set to $\delta = 0$ ppm, with internal lock signals tuned using D_2O. NMR spectral data were processed using Mnova version 5.1 (Mestrelab Research). Electrospray ionization mass spectra (ESI-MS) were recorded from a LTQ Orbitrap XL instrument (Thermo Scientific, with an accuracy of 0.1 amu) with a nano spray ion source. Tetrabutylammonium salts (TBA cation) of the POMs were used to make a solution in pure acetonitrile and directly injected for the ESI-MS measurements. Infrared (FTIR) spectra were acquired from KBr pellets using a Nicolet Impact 410 spectrophotometer. Cyclic voltammetry (CV) of the POMs and iron-POM molecular complexes and Differential Pulse Voltammetry (DPV) of the POM-complexed cores were carried out in a three electrodes cell set up on a CHI 760C potentiostat at 25 ± 2 °C in 0.2 M $LiClO_4$ electrolyte solutions, using a 2-mm glassy-carbon, Pt-wire and Ag/AgCl (3M KCl) working, counter and reference electrodes respectively. Raman spectra of POMs and POM-complexed iron-oxide NCs were recorded with a Horiba−Jobin−Yvon Lab Ram HR 800 micro-Raman system, equipped with a Synapse CCD detector using a laser excitation source (515 nm) and ND filters. Powder-XRD data were obtained using a Panalytical Empyrean instrument using Cu Kα radiation (λ = 1.5405 Å), operated at 40 kV and 30 mA, and equipped with a position sensitive (PSD) X'Celerator detector. DLS data was collected at 25 °C on an ALV-CGS-8F instrument (ALV-GmbH, Germany) at 90 deg (unless otherwise indicated), and the CONTIN method was used to obtain hydrodynamic radii (R_h). Zeta potentials were measured using a ZEM 3600 Zetasizer (Malvern Instruments, Ltd). Transmission electron microscopy (TEM) images of the iron-oxide nanoparticles were taken in a FEI Tecnai 12 G2 electron microscope (120kV) equipped with a Gatan slow-scan camera. High resolution transmission electron microscopy (HRTEM) images were taken in a JEOL JEM-2100F instrument operated at an accelerating voltage of 200 kV. Scanning TEM (STEM-HAADF) images were collected using an annular dark-field JEOL detector (EM 24560). EDS analyses were done using a JEOL JEM- 2100F TEM operating at 200 kV equipped with a JED-2300T energy dispersive X-ray spectrometer. JEOL Analytical Station software (v. 3.8.0.21) was used for the EDS data analysis. Cryogenic-TEM images were taken on the FEI Tecnai 12 G2 instrument (120kV) and the Gatan slow-scan camera, using a low-dose regime to slow down the crystallization of vitrified water and to delay the formation of other artifacts due to beam damage. Samples for cryo-TEM imaging were prepared using a fully automated vitrification device ("Vitrobot"). All images from both dry- and cryo-TEM (including electron diffraction patterns) were analyzed using Digital Micrograph Gatan Inc. software. X-ray photoelectron spectroscopy (XPS) of POMs and the hybrid iron-oxide NCs were performed using an X-ray photoelectron spectrometer ESCALAB 250 ultrahigh vacuum ($1 \times 10{-}9$ bar) apparatus with an AlKα X-ray source and a monochromator with an X-ray beam size 500 µm. The survey spectra were recorded with pass energy (PE) 150 eV and high energy resolution spectra were recorded with pass energy (PE) 20 eV. All the XPS results were processed using AVANTGE program. Oxygen evolved during photochemical reactions was quantified using a Thermo Scientific Focus Gas Chromatograph (GC). The GC was equipped with a dedicated thermal-conductivity detector (TCD), Ar was used as the carrier gas, and peak areas were calibrated using air as the calibration standard (20.9 % O_2 in air).

Isolation and purification of 1. The nano-sized POM-complexed α-Fe_2O_3 was first precipitating by addition of NaCl (to a final concentration of 2 M). Under these conditions, *reversible* aggregation of the **1** nanocrystals (NCs) decreases their solubility in water, so that they can be separated from the supernatant solution by centrifugation (30 min at 6000 rpm). Notably, millimolar concentrations of the primary POM by-products, are fully soluble in 2 M NaCl, and remain in the supernatant solution. After decanting the supernatant solution by pipette, **1**, a hydrated dark-red solid, was collected and dissolved in 10 mL of pure water, to give a clear, orange-red solution. Two additional "washing" cycles of precipitation by addition of NaCl, followed by centrifugation and re-dissolution in pure water, were carried out to be certain that trace amounts of byproducts were no longer present. This removed a significant amount of the polyoxometalate

by-products, which consisted primarily of phosphate (see Supplementary Fig. 2 for ^{31}P NMR spectra and the balanced equation for the overall reaction), iso-polytungstates and POM byproducts. Any remaining traces of byproducts still present in the solution, along with small amounts of NaCl precipitated out with the nanostructures, were removed by dialysis for 24 h against pure water (1 L). This involved placing the solution in a cellulose membrane in a 2 L beaker, during which time, the water outside the dialysis membrane was replaced every 12 hours. (Note: after just two precipitation cycles and dialysis, the amount of "free" POM byproducts present in the supernatant solution was already below its detection limit by UV spectroscopy, i.e., less than 2 μM.).

Characterization of 1. Characterization of the hematite-Fe_2O_3 core was achieved by routine microscopic tools in solid state while the POM ligands on the particle's surface were characterized by a variety of solution-state microscopic and spectroscopic techniques and analytical tools. The characterization of POM ligands on the surfaces of colloidal α-Fe_2O_3 nanocrystals in solution is extremely challenging. Recently covalently attached POMs on TiO_2 cores were fully characterized using a variety of solid- and solution-state microscopic, diffraction, and spectroscopic methods, combined with chemical etching, cation- and (*attempted*) anion-exchange experiments, and electrochemistry.[8] After mild etching of the surface of **1** with dilute HCl, the POM ligands attached to the surface of the NCs were characterized by UV-vis spectroscopic and ESI-Mass analysis. For this, three individual reaction solutions of **1** (10 mL each; prepared and purified as described above) were treated at room temperature with 1M HCl to reach a final pH value 2. After gently stirring the orange-red solution for 4 h at 60-70 ^{o}C, a cloudy orange-red solution resulted. The orange-red precipitate was spun down by centrifugation and the supernatant was then removed by pipette. The absorbance observed in the UV region by UV-vis spectroscopy was consistent with the presence of "[α-$PW_{11}O_{39}Fe]^{4-}$" (see Supplementary Fig. 12) that is, comparable to the charge transfer band observed in the UV for independently prepared [α-$PW_{11}O_{39}Fe(X)]^{4-}$ (X = H_2O).[2,3] To the supernatant solution obtained after etching, 1 mL of a 100 mM aq solution of TBABr was added. The solution soon became cloudy and a pale-yellow solid was isolated by centrifugation. After washing several times with pure water, the solid was dissolved in CH_3CN and used for ESI-Mass analysis.

Characterization of the reaction byproduct, [α-$PW_{11}O_{39}Fe(OH)]^{5-}$. The ^{31}P NMR spectrum of the reaction solution exhibits free phosphate (Supplementary Fig. 2) formed by base hydrolysis of POM during heating at 220 ^{o}C at pH 6.7. This is expected, as the POM polishes the amourphous iron oxide by complexing with Fe(III) atoms, a process that liberates oxide or hydroxide. At the same time, ^{31}P signals related to the POM was not seen. That is, the decanted solution obtained after precipitation of **1** by addition of NaCl contains Fe(III)-substituted byproduct, [α-$PW_{11}O_{39}Fe]^{4-}$, but no ^{31}P signal was observed. This is due to the presence of paramagnetic Fe(III) at the lacunary site of [α-$PW_{11}O_{39}]^{7-}$. UV-vis spectra of the decanted NaCl solution, however, shows a sharp absorbance near 250 nm, consistent with the POM (Supplementary Fig. 3). And, cyclic voltammograms of the supernatant solutions exhibit two reversible redox waves at -653 mV and -820 mV, characteristic for sequential one-e- reductions of W(VI), along with a reversible redox feature for the Fe(III/II) couple at 50 mV (Supplementary Fig. 4). After isolating a small amount of the byproduct by addition of TBA-Br, the FTIR spectrum (Supplementary Fig. 6a) was identical to that of pure $(TBA)_5[PW_{11}O_{39}Fe(OH)]\cdot 2H_2O$[7]; FTIR (KBr): γ_{as}(P-O) 1070, 1060 cm^{-1}, γ_{as}(W=O) 955 cm^{-1}, γ_{as}(W-O-W, inter-triad corner-sharing) 877 cm^{-1}, and γ_{as}(W-O-W, intra-triad edge-sharing) 810 cm^{-1}. ESI-Mass spectra of the acetonitrile solution of the TBA-salt isolated from the reaction mixture shows a predominant molecular-ion peak at m/z = 3702.33, matching the simulated molecular-ion peak for $\{(TBA)_4H[\alpha\text{-}PW_{11}O_{39}Fe]\}^{+}$ (Supplementary Fig. 5). A balanced equation (given below) has been derived after quantification of byproducts formed during the hydrothermal reaction.

Preparation of organic-solvent soluble *n*-R_4N^+ substituted 1. To the purified solution of **1**, *n*-$Hex_4N^+Br^-$ (THABr) was added to a final concentration of 5 mM, giving an orange precipitate that was collected by centrifugation and washed three times with pure water. Upon addition of MeOH (10 mL), the orange solid dissolved completely, giving an optically clear solution of the organic-solvent soluble *n*-Hex_4N^+ salt of **1**.

DLS results and TEM data are provided in Supplementary Fig. 15. A similar method was used to prepare TBA and *n*-Octyl$_4$N$^+$ salts of **1**, soluble in MeCN and MeOH.

Calculation of quantum efficiency for photochemical O_2 production. A 300 W Xe arc lamp, equipped with 450 nm band pass filter light source was used to irradiate the quartz cell contain an aq solution of **1** and periodate. The band pass filter produced a single wavelength of blue light (λ = 450 nm). The intensity of the blue light was measured by 1928-C Optical Power Energy Meter (Newport Corp.) equipped with a model 919P-250-35 Thermopile sensor. The light intensity was ca. 2.55 mW/cm^2 on the sample with an area of illumination of 1 cm^2. The number of absorbed photons was assumed to be equivalent to the difference in the number of incident photons and the photon transmitted out from the quartz cell. For the cloudy-orange solution of 5 nm α-Fe_2O_3,[6], no transmitted photons were detected by the power meter, while for the clear solution of **1**, a negligible loss of light intensity was calculated by subtracting the light intensity transmitted by the solution from the incident intensity.

The energy of each photon of 450 nm blue light is 4.41 x 10^{-19} j/photon (E of photon = hc/λ; where h is Planck's constant, c is the speed of light and λ wavelength of light). Under our experimental conditions, the intensity of the incident light is 2.55 mW, and the photon flux (number of photons per sec) is defined as the intensity of incident light divided by the energy of a photon. Hence, photon flux is equal to 5.78 x 10^{15} (Photons/s), that is, equivalent to 9.60 x 10^{-9} (Einstein/s). Quantum yield is defined as the rate of photochemical O_2 production with respect to the absorbed photon per unit time. Since, each oxygen molecule production requires four electrons, and therefore four photons, the quantum yield for oxygen production was determined by the following equation:

QE (Φ_{O2}) = 4·(rate of O_2 production, mol·s^{-1}) / absorbed photon per unit time (Einstein·s^{-1}).[9]

Photochemical reaction in MeCN and MeCN-H_2O (1:1) mixture. The photochemical reaction in MeCN was performed with the n-Hex$_4$N$^+$ salt of **1** (prepared as described above) in the presence of 20 mM tetrabutylammonium periodate (TBAIO$_4$; freshly prepared by addition of TBAHSO$_4$ to the aq solution of NaIO$_4$) in gas tight quartz cell with 16 mL headspace volume. To the MeCN solution of the THA-salt of **1**, solid TBAIO$_4$ was added, and the solution was purged with Ar (g) for 20 minutes and then irradiated for 8 h using a 150 W Xe lamp (USHIO inc. Japan) with a 400-nm filter ($\lambda \geq$ 420 nm). The headspace gas was injected into the GC at 2 h intervals. The reaction in 1:1 MeCN-H_2O was carried out using a TMA salt of **1**, and TBAIO$_4$.

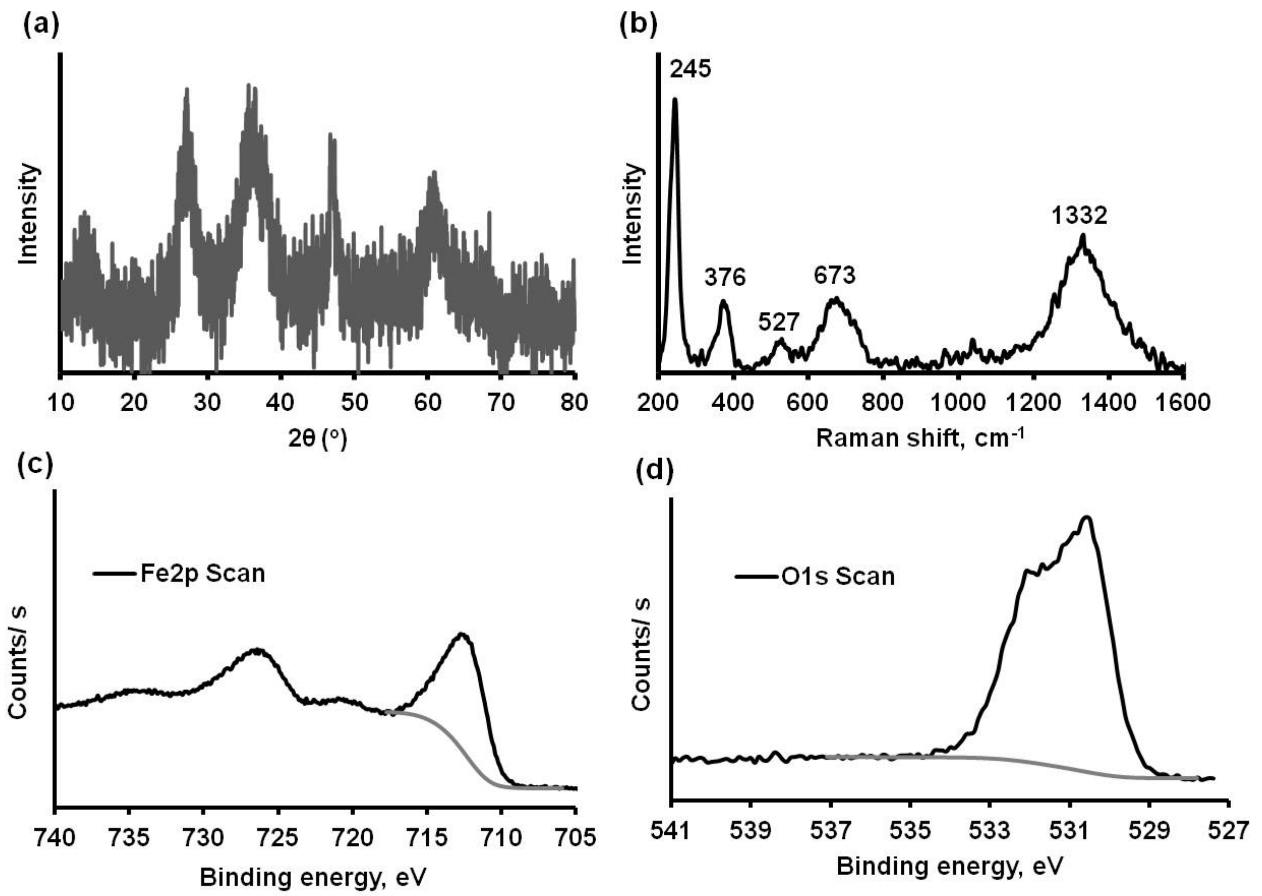

Supplementary Figure 1: Characterization of the orange suspension of micron-sized particles obtained after reaction of $FeSO_4$ with KOH in air (a) Powder X-ray diffraction of the orange residue is very broad and exhibits a diffraction pattern similar to γ-FeOOH (s). Four broad peaks within 20°–70° (2θ range) were indexed to hkl value; 011, 031, 060 and 211 by comparing XRD pattern of reported γ-FeOOH in literature. (b) The orange suspended solid shows major Raman shifts in between 200-800 cm^{-1} the position of the bands matches well with γ-FeOOH. (c) A high-resolution XPS analysis for Fe2p and (d) high-resolution XPS analysis for O1s. Position of the Fe2p scan corresponds to iron(III) while O1s indicates the presence of oxide (O^{2-}) and hydroxide (OH^-) with a 50:50 ratio of peaks. All the spectroscopic and analytical data of the orange solid isolated upon reaction of aq $FeSO_4$ with 2.2 equiv of KOH in presence of air, support the formation of micron size partially amorphous γ-FeOOH (s).

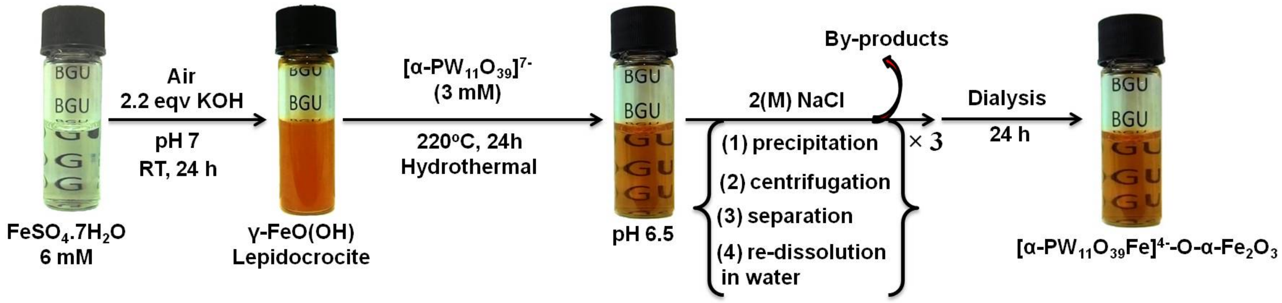

Supplementary Figure 2: Addition of 2.2 equivalent of KOH to the pale-yellow solution of $FeSO_4$ results in formation of a cloudy-orange suspension of γ-FeO(OH) (s) with a pH value of 7. Then, heating that solution at 220 °C for 24 h in presence of a half an equivalent of $[\alpha\text{-}PW_{11}O_{39}]^{7-}$ gave an optically transparent orange solution with a pH value of 6.5. Addition of 2 M NaCl resulted in orange precipitate of the POM-complexed hematite NCs, leaving water-soluble byproducts in the supernatant. Centrifugation, followed by separation and re-dissolution in water, resulted in purified nanostructures. The "washing" step was followed thrice in order to remove most of the soluble molecular POMs and inorganic salts. Traces of byproducts and residual NaCl were then removed by dialysis for 24 h.

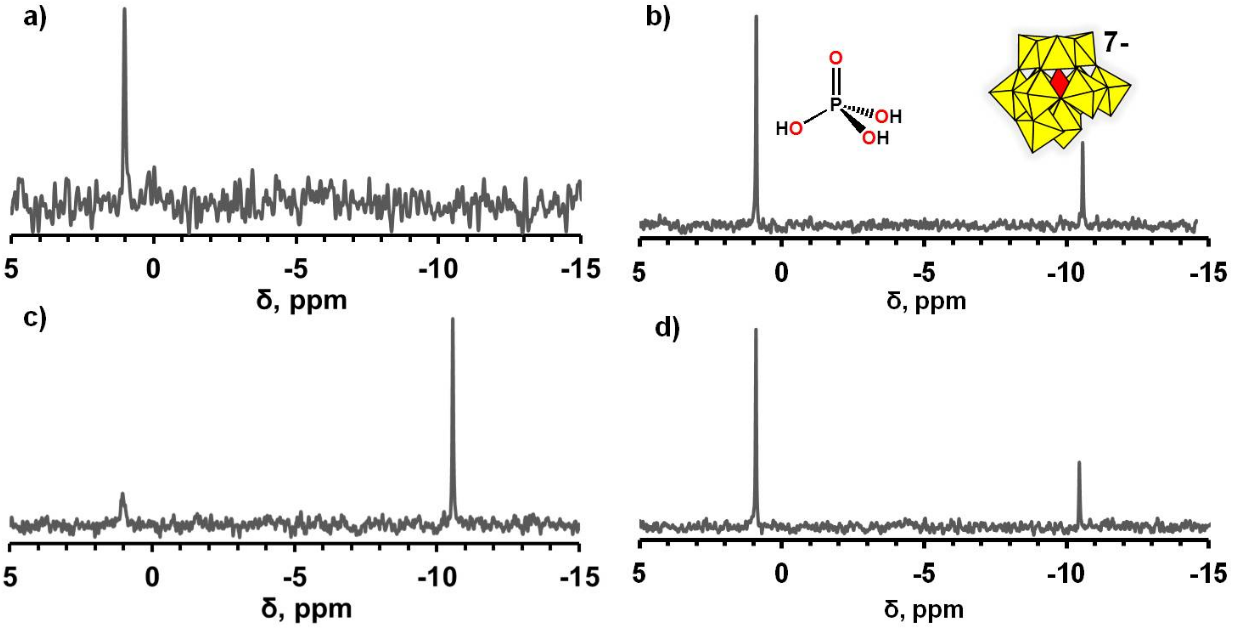

Supplementary Figure 3: Characterization of byproducts by ^{31}P NMR; (a) ^{31}P NMR spectrum of the solution obtained after reaction of amorphous γ-FeOOH with $Na_7[\alpha\text{-}PW_{11}O_{39}]$ for 24 h at 220 °C (see Experimental Methods; the reaction results in formation of free phosphate). (b) P-31 NMR spectrum of a mixture of *o*-phosphoric acid and $[\alpha\text{-}PW_{11}O_{39}]^{7-}$ (ratio 2:1), (c) P-31 NMR of the 250 μL reaction solution and 250 μL of 3 mM $[\alpha\text{-}PW_{11}O_{39}]^{7-}$ in the presence of 50 μL of D_2O; (d) ^{31}P NMR of the 250 μL reaction solution and 250 μL of mixture *o*-phosphoric acid and $[PW_{11}O_{39}]^{7-}$ (ratio 2:1) in presence of 50 μL of D_2O. In panel b, *o*-phosphoric acid appeared at 0.91 ppm, while $[\alpha\text{-}PW_{11}O_{39}]^{7-}$ appear at -10.52 ppm, the intensity of the peaks is 2:1. Panel c represents that addition of $[PW_{11}O_{39}]^{7-}$ to the reaction solution doesn't change the peak at 0.91 ppm. Comparison of panels **a**, **b** and **c** indicates the formation of free phosphate during the reaction of reaction of amorphous γ-FeOOH with $[PW_{11}O_{39}]^{7-}$ for 24 h at 220 °C. Panel (**d**) represents the quantification of free phosphate (0.91 ppm) formed during the reaction of γ-FeOOH with $[PW_{11}O_{39}]^{7-}$ with respect to the 2:1 mixture of *o*-H_3PO_4 and $Na_7PW_{11}O_{39}$. The ratio of the integrated peak intensities of phosphate and the pure POM suggests that ca. 20% free phosphate is forming during the reaction.

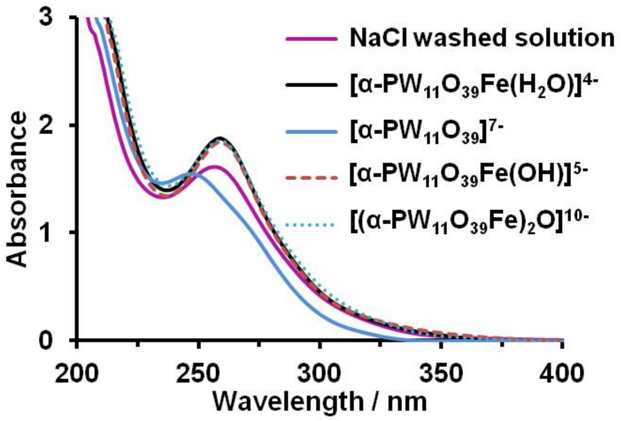

Supplementary Figure 4: UV-vis spectra of the pale-yellow supernatant obtained from 2 M NaCl treatment of the optically clear orange-red reaction solution of **1**. NaCl-washed supernatant was diluted before recording the optical spectra (60 times dilution with water) and compared with the UV-vis spectra of 50 μM of $Na_4[\alpha\text{-}PW_{11}O_{39}Fe(H_2O)]$, $Na_7[\alpha\text{-}PW_{11}O_{39}]$, $Na_5[\alpha\text{-}PW_{11}O_{39}Fe(OH)]$ and $Na_{10}[(\alpha\text{-}PW_{11}O_{39}Fe)_2O]$. Only $Na_7[\alpha\text{-}PW_{11}O_{39}]$ shows an absorption maximum at 248 nm, while NaCl washed supernatant and all iron-complexes of $[\alpha\text{-}PW_{11}O_{39}]^{7-}$ exhibit absorption maxima at 258 nm. The red shift in absorption maxima of the NaCl-washed supernatant solution is consistent with incorporation of Fe(III) into the lacunary site of $[\alpha\text{-}PW_{11}O_{39}]^{7-}$. A comparison of the intensity of the CT band at 258 nm reveals ca. 2.0 mM iron-complexed $[\alpha\text{-}PW_{11}O_{39}]^{7-}$ in the decanted NaCl washed solution.

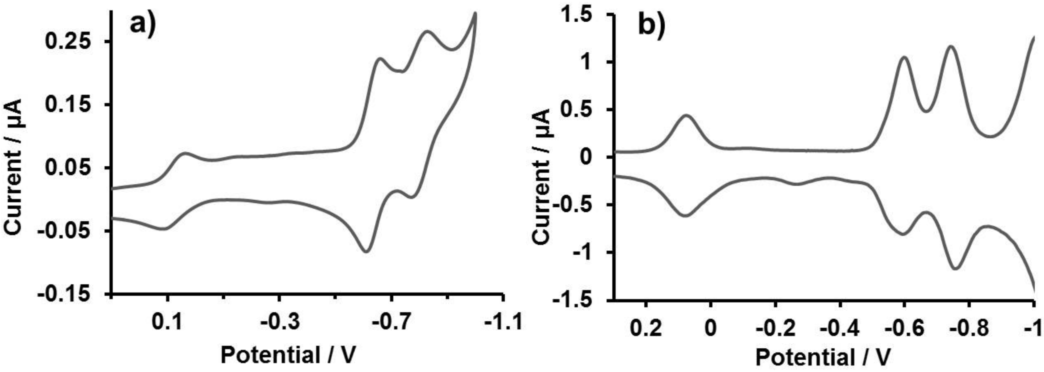

Supplementary Figure 5: Cyclic voltammetry and DPV of the decanted 2 M NaCl solution left after centrifugation of the orange-red precipitate of salted-out **1**; (a) cyclic voltammogram of the decanted 2 M NaCl solution in aqueous acetate buffer of pH 3.3, (b) forward and reverse differential pulse voltammograms of the same solution under similar condition. Three reversible redox waves (76 mV, -596 mV and -744 mV) in the cyclic voltammogram, and in the forward and reverse DPVs of the NaCl-washed solution are consistent with $[\alpha\text{-}PW_{11}O_{39}Fe]^{4-}$. The reversible feature at 100 mV was assigned to a the Fe(III/II) couple, while the other two sharp features in the cathodic region, at -595 mV and -777 mV, are consistent with sequential one-electron reductions of W(VI) atoms of the POM.

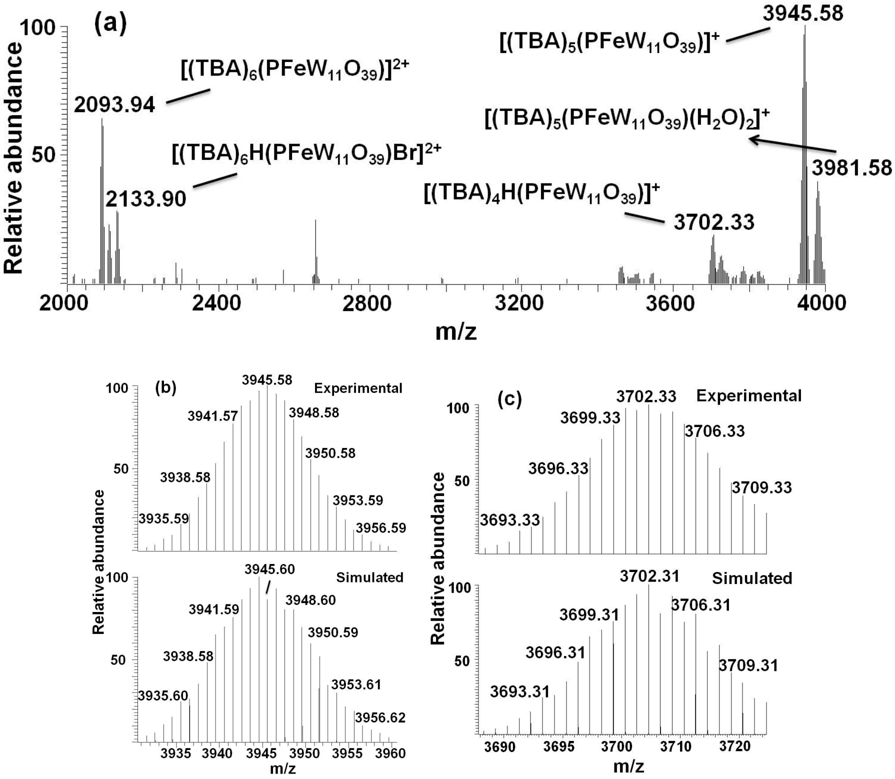

Supplementary Figure 6: ESI mass spectrum of a MeCN solution of the pale-yellow solid obtained upon addition of $(n\text{-}Bu_4N)Br$ to the decanted 2 M NaCl solution obtained during purification of **1**. (a) ESI-mass spectrum indicates formation of Fe-POM complex. All the major peaks match with the differently hydrated complexes of TBA-$[PW_{11}O_{39}Fe]^{+}$. (b) and (c) are isotopic distributions of the major peaks at m/z = 3945.58 and 3702.33, respectively, in comparison to their simulated spectra.

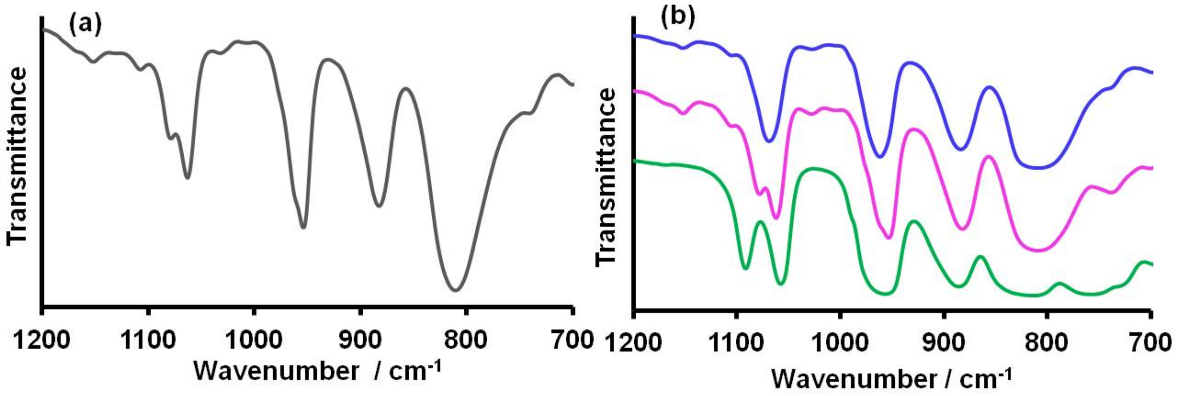

Supplementary Figure 7: (**a**) FTIR spectrum of the pale-yellow solid obtained upon addition of (n-Bu_4N)Br to the decanted 2 M NaCl solution during purification of **1**. The FTIR spectrum of the pale-yellow solid exhibits an asymmetric P-O stretching band with small split, (Δv 10 cm^{-1}), along with other typical W=O stretching bands between 1000-800 cm^{-1}. Meanwhile, splitting of the P-O stretching bands of the pale-yellow solid differ from independently prepared $TBA_{10}[(\alpha\text{-}PW_{11}O_{39}Fe)_2O]$ (**b**, green curve) and $TBA_4[\alpha\text{-}PW_{11}O_{39}Fe(H_2O)]$ (**b**, purple curve). Notably, however, the Δv value for the P-O stretching is 12 cm^{-1} in $TBA_5[\alpha\text{-}PW_{11}O_{39}Fe(OH)]$ (**b**, pink curve). These spectra provide another line of support for formation of $[\alpha\text{-}PW_{11}O_{39}Fe(OH)]^{5-}$ as a byproduct during reaction of γ-FeOOH with $Na_7[\alpha\text{-}PW_{11}O_{39}]$.

Supplementary Discussion (Balanced equation): P-31 NMR spectra of the as-obtained reaction solution reveals 20% of $Na_7[\alpha\text{-}PW_{11}O_{39}]$ ca. 0.6 mM (starting with 3 mM POM solution) is hydrolized to form free phosphate. Reaction of 6 mM ferrous sulphate solution with 3 mM POM would yield a maximum of 3 mM (considering 100% yield) of $[\alpha\text{-}PW_{11}O_{39}Fe(OH)]^{5-}$. Comparing the relative intensity of the absorption maxima at 258 nm, about 80% $[\alpha\text{-}PW_{11}O_{39}Fe(OH)]^{5-}$ is present in NaCl washed supernatant. A balanced equation for the reaction of γ-FeO(OH) (s) with POM yielding 4: 1 ratio of $[\alpha\text{-}PW_{11}O_{39}Fe(OH)]^{5-}$ and free phosphates is thus:

$$4\,[\gamma\text{-FeO(OH)}] + 5\,[\alpha\text{-}PW_{11}O_{39}]^{7-} \xrightarrow{H_2O} 4\,[\alpha\text{-}PW_{11}O_{39}Fe(OH)]^{5-} + \tfrac{1}{2}\,[HPO_4]^{2-} + \tfrac{1}{2}\,[H_2PO_4]^{1-} + \frac{11}{7}\,[W_7O_{24}]^{6-}$$

Starting with 6 mM amorphous γ-FeO(OH)(s) (considering 6 mM $FeSO_4\cdot 7H_2O$ was converted to 6 mM γ-FeO(OH)(s)) and 3 mM $Na_7[\alpha\text{-}PW_{11}O_{39}]$, the final concentration of by-product $[\alpha\text{-}PW_{11}O_{39}Fe(OH)]^{5-}$ is 2.4 mM. The amount of POM remaining bound to the iron-oxide core is much smaller (see Supplementary Table 1).

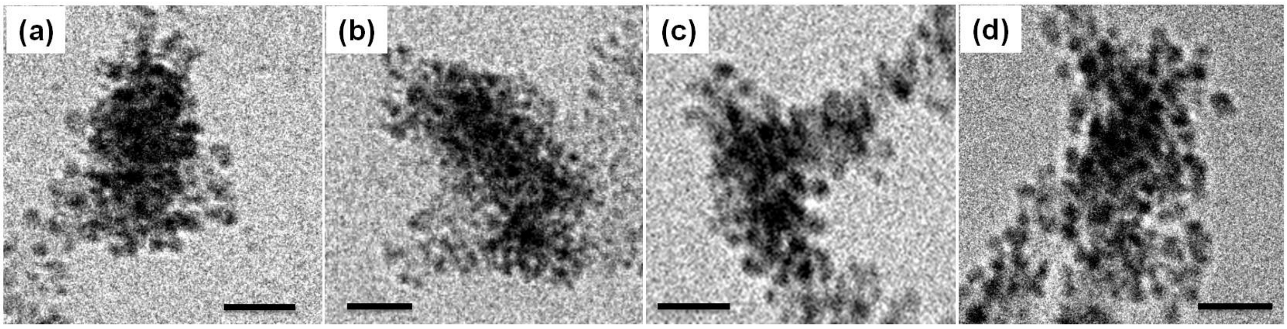

Supplementary Figure 8: Cryo-TEM images of small aggregates of **1** (scale bars: 10 nm). The individual hematite cores of **1** have been shown in the main text to be ca. 3 nm in diameter (see Fig. 1b in the main text). As noted in the text, individual POMs are not expected to be imaged on very small (3 nm) particles. Apart from images of those individual particles (Fig. 1b of the text), small aggregates can also be found in cryo-TEM images. The dark ca. 1-nm objects on the small aggregated structures are likely to be heteropolytungstate cluster anions. However, due to the small size of the individual cores, and the comparable size of the POM, it is very difficult to be entirely certain that the small dark (1 to 2 nm) objects in the slightly aggregated structures are POM ligands. For this reason, we cannot claim that the small dark objects observed here are, in fact, the POM ligands. At the same time, other spectroscopic data and control experiments show that, similar to POM-complexed-TiO_2 NCs,[9] the POM ligands are present and covalently attached to the Fe_2O_3 cores.

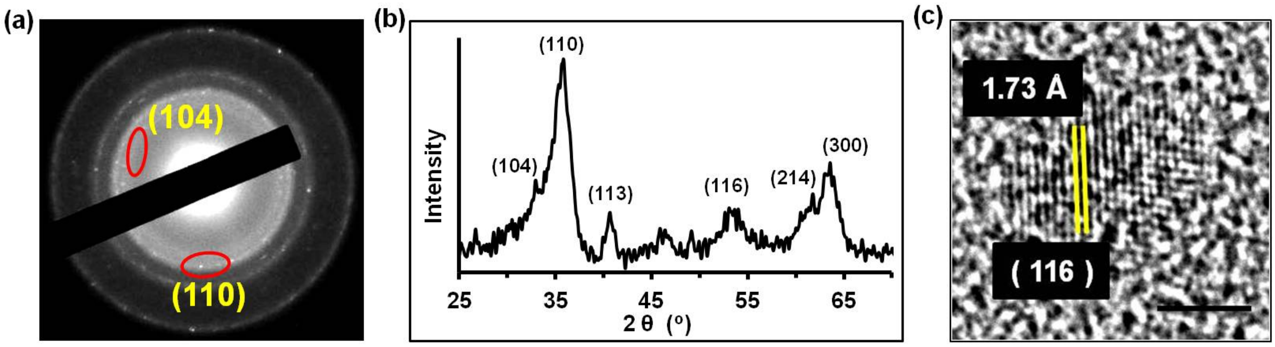

Supplementary Figure 9: Characterization of hematite-Fe_2O_3 cores in **1**; (a) Electron diffraction of the selected area of **1** reveals well defined ring of (104) and (110), diagnostic for hematite nanocrystals, (b) Powder X-ray diffraction of the hematite cores. The size of the hematite-Fe_2O_3 crystallites (3.5 ± 0.5 nm) was determined using the Debye-Scherrer's equation, $L = 0.9\lambda/\beta \text{Cos}\Theta$, where, L is the size of the crystallites, λ is the X-ray wavelength (nm), β is the peak width at half maximum (in radians) of the most intense diffraction peak, and θ is the Bragg angle of diffraction. (c) HRTEM images of purified **1** showing an individual NC core with well-defined lattice fringes from (116) planes (scale bar: 2 nm).

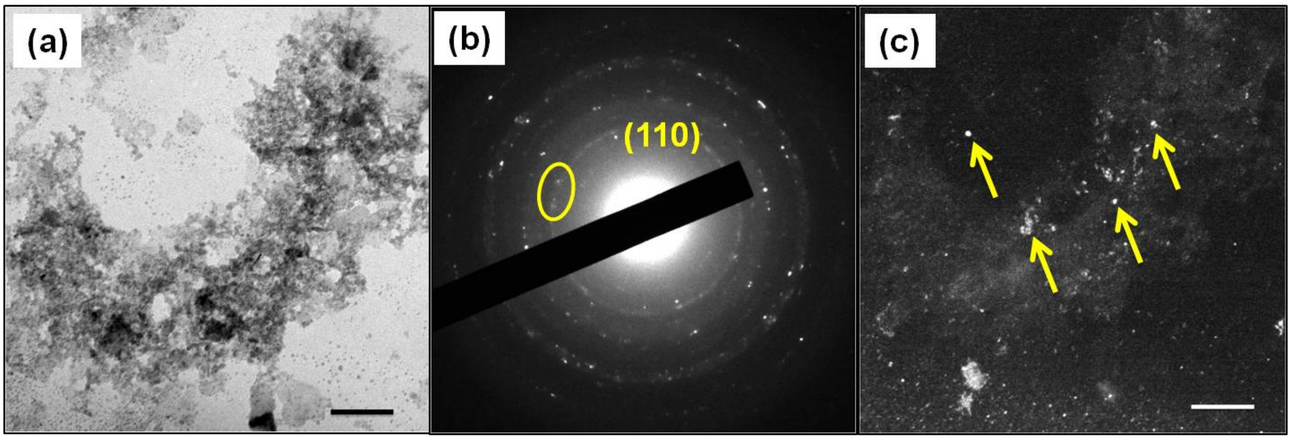

Supplementary Figure 10: Characterization of hematite-Fe_2O_3 cores in **1**. (a) Brightfield-TEM image of the purified dried **1** NCs (scale bar: 100 nm). (b) Selected area electron diffraction pattern of the particles in panel (a). (c) Darkfield-TEM image (scale bar: 100 nm) obtained from the yellow-encircled part of the (110) ring in panel (b). Here, diffraction from similarly oriented (110) reflection planes (yellow-encircled area in panel b), was used to selectively "illuminate" (and image) individual nanocrystalline cores of **1** (yellow arrows in panel c).

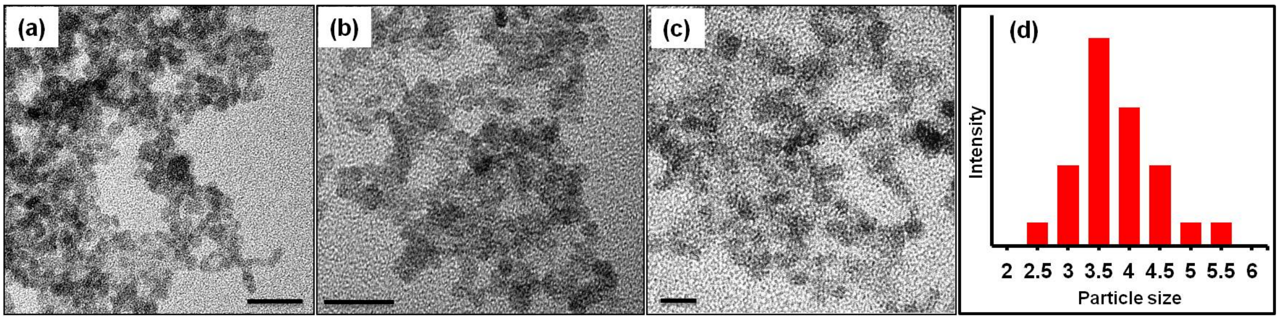

Supplementary Figure 11: TEM images of the dried **1** and particle-size distribution. (a)-(c) TEM images of purified **1**, and (d) particle-size distribution (scale bars: 20 nm in panel a and b; 10 nm in panel c). The dried nanostructures appeared as ca. 3.5 nm (average-diameter) particles, which is in line with the average core size calculated using the Scherrer equation to evaluate the powder X-ray diffraction data from the material.

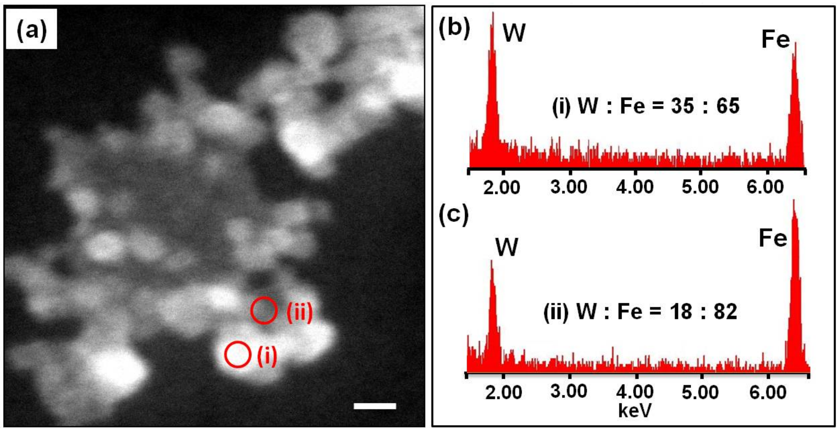

Supplementary Figure 12: High angle annular dark field (HAADF) images and energy dispersive X-ray (EDX) spectra of **1**. (a) HAADF image of several **1** nanostructures, grouped together in the solid state (scale bar: 5 nm). (Due to the small size of the NC cores, individual POM ligands are not well resolved in the HAADF image). (b) EDX spectra (taken using a small 0.2 x 0.2 nm^2 spot size) of the red circle labelled “i” in panel “a” which indicates a relatively large W content, and (**c**) corresponds to the red circle “ii” in panel a, a location relatively poor in W. This confirms that the brighter areas contain the heteropolytungstate cluster-anions, whereas the less-bright areas are predominantly Fe_2O_3.

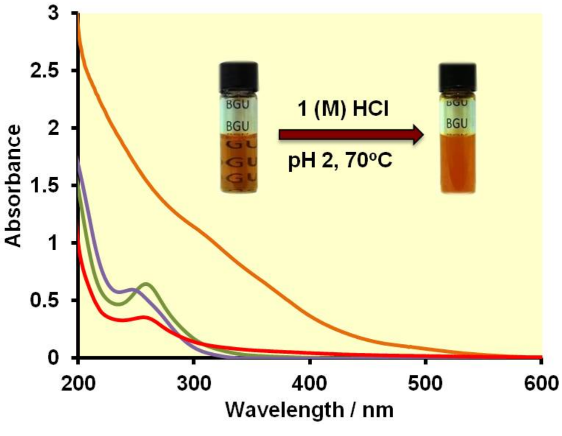

Supplementary Figure 13: Etching the surface of **1** and detection of the liberated POM ligand by UV-vis spectroscopy. The orange curve is for **1**, the green curve is for $[\alpha\text{-}PW_{11}O_{39}Fe(H_2O)]^{4-}$, prepared independently, the purple curve is of $[\alpha\text{-}PW_{11}O_{39}]^{7-}$, and the red line is the supernatant solution after mild HCl etching of **1** from the hematite-cores (as described above). The spectrum of the supernatant solution after etching (and liberation of POM ligands into bulk solution—red curve) is comparable (λ_{max} = 258 nm) to solutions of pure $[\alpha\text{-}PW_{11}O_{39}Fe(H_2O)]^{4-}$.

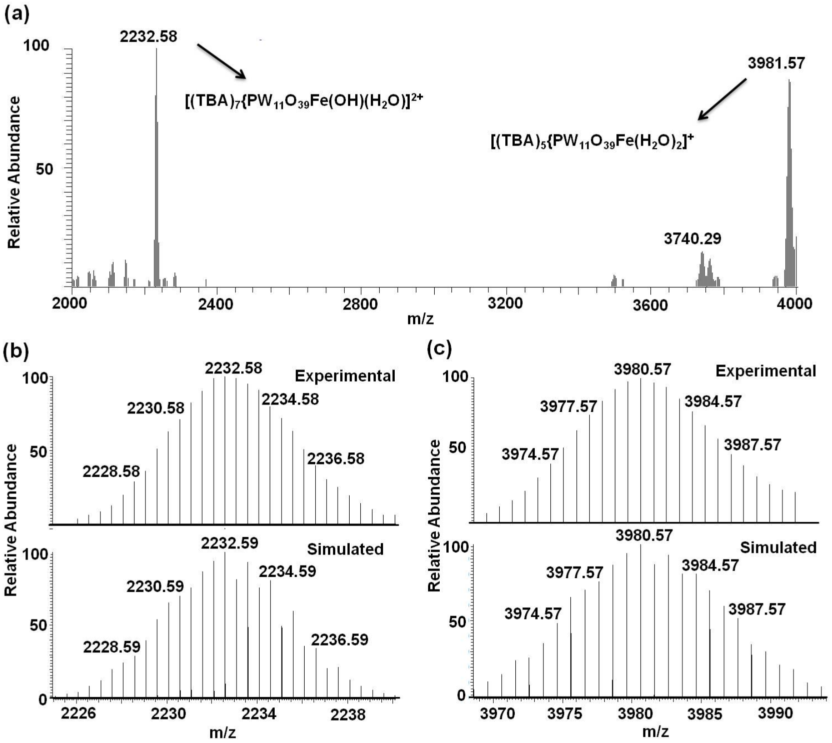

Supplementary Figure 14: Etching the surface of hematite-Fe_2O_3 cores in **1** and characterization of the POM ligand by ESI-Mass. (a) ESI-mass spectrum of a MeCN solution of the residue obtained after mild etching followed by precipitation of the liberated POM by the addition of TBABr. Two intense signals at m/z = 2232.58 and 3981.57, respectively are observed. (b) Isotope distribution pattern of the experimentally observed ion peak at m/z = 2232.58 (top) and the simulated pattern obtained for molecular ion $[(TBA)_7\{PW_{11}O_{39}Fe(OH)(H_2O)\}]^{2+}$ (bottom). (c) Isotope distribution pattern of the experimentally observed ion peak at m/z = 3980.57 (top), and the simulated pattern obtained for molecular ion $[(TBA)_5\{PW_{11}O_{39}Fe(H_2O)_2\}]^{+}$ (bottom).

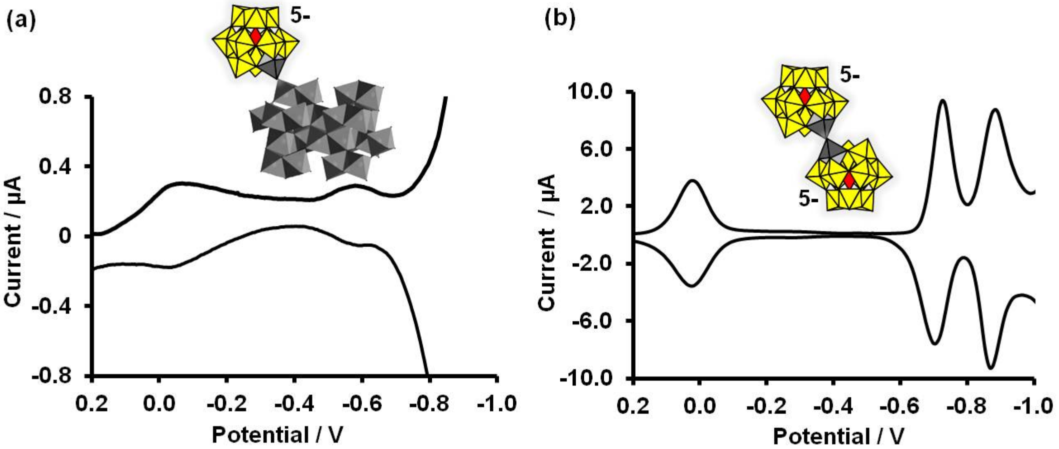

Supplementary Figure 15: Differential pulse voltammograms (DPVs). (a) DPV of **1** in presence of 100 mM $LiClO_4$ in aq acetate buffer at pH 3.3, and (b) DPV of the dimeric molecular iron-POM complex, $[(PW_{11}O_{39}Fe)_2O]^{10-}$ in the presence of 100 mM $LiClO_4$ at pH 5. Two reversible redox waves (at -50 mV and -590 mV) in the DPV of the material in panel (a) correspond to the Fe(III/II) redox couple, and to 1-e^- reduction of W(VI) in $[\alpha\text{-}PW_{11}O_{39}Fe]^4$ on the surface of the hematite cores, while three redox processes are observed for $[(PW_{11}O_{39}Fe)_2O]^{10-}$, consistant with a reversible Fe(III/II) couple and sequential one-electron reductions of W(VI). Notably, in panel (a) the second (more negative) redox couple of POM W(VI) atoms is not well resolved due to overlap with a large cathodic current at -700 mV (possibly due to H_2 formation, and/or to reduction of Fe(III) in the hematite cores).

Supplementary Table 1: Calculation of atom-fractions of Fe and W of **1**, considering a 3.0 nm (Diameter) ideal spherical α-Fe_2O_3 NC core and 1.9 nm^2 POM footprint on the hematite surface. The atom-fractions of Fe and W vary by ± 6% (corresponding to the ± 5% uncertainty based on different EDX measurements).

[Fe(III)] (as α-Fe_2O_3)	0.0036 M
Density of α-Fe_2O_3	5.24 x 10^{-21} g nm^{-3}
Mol. Wt. of α-Fe_2O_3	159.69 g mol^{-1}
Volume of α-Fe_2O_3 solution	0.01 L

Moles of Fe(III)	3.60×10^{-5} mol
Mass of α-Fe_2O_3 in 0.01 L solution	2.87×10^{-3} g
Volume of α-Fe_2O_3	5.49×10^{17} nm^3
Volume of one α-Fe_2O_3 NC[a]	14.13 nm^3
**No. of Fe atoms per NC**	**279**
No. of α-Fe_2O_3 NCs	3.88×10^{16}
[α-Fe_2O_3] NCs	6.446×10^{-6} M
Surface area of one α-Fe_2O_3 NC	28.26 nm^2
POM footprint on NC surface	1.9 nm^2
[POM] (bound to NCs)	95.9 μM
**No. of POMs per NC**	**15**
Sum of W + Fe atoms / particle	4.43×10^2
**Atom/atom fraction of Fe[b]**	**0.63**
**Atom/atom fraction of W[b]**	**0.37**

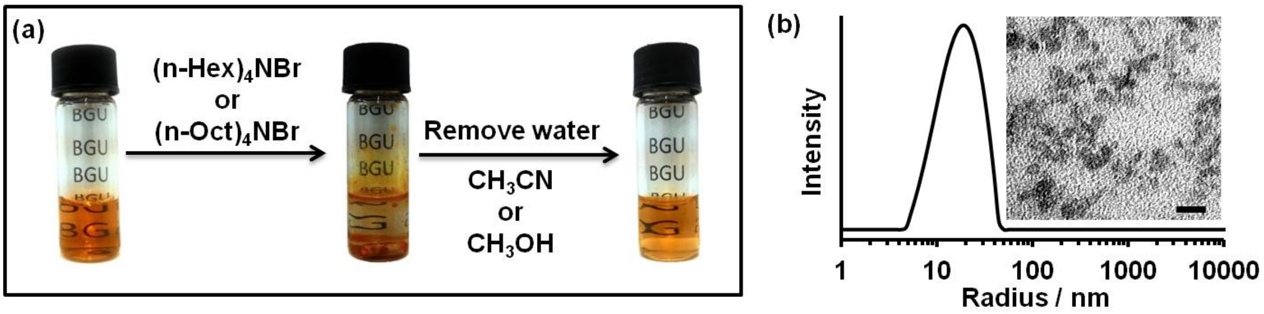

Supplementary Figure 16: Preparation and characterization of tetra-alkylammonium cation salts of **1**. (a) Images of a vial containing an aq solution of **1** (Na^+ salt; left), which is precipitated by addition of excess Br^- salts of *n*-hexylammonium or *n*-octylammonium cations. After removing the nearly transparent supernatant, the residual material was dissolved in CH_3CN or CH_3OH. (b) DLS data (unweighted) of a MeCN solution of the *n*-Hex_4N^+ salt of **1**. (b, inset) TEM images of the dried MeCN solution consistent with small particles of **1** (scale bar: 10 nm).

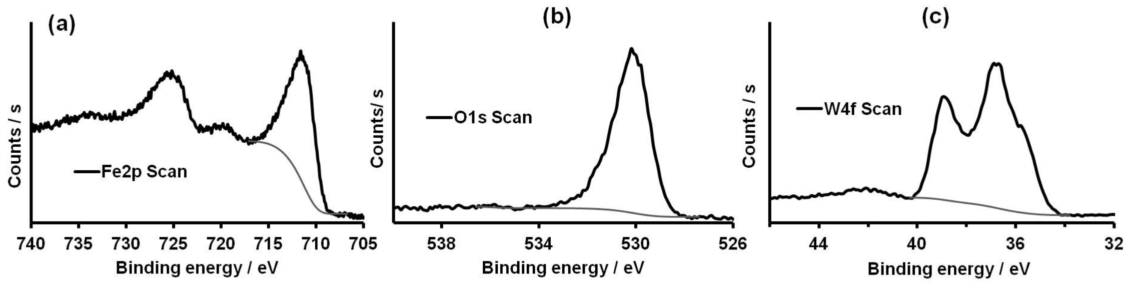

Supplementary Figure 17: High-resolution XPS spectra showing that **1** is comprised of Fe, O and W. (a) High-resolution Fe2p core-level spectrum indicates Fe(III) atoms. (b) High-resolution O1s core-level spectrum. The O1s scan matches with a majority of O^{2-} ions in the NC core, and few H_2O ligands possibly adsorbed on the hematite surface. (c) High-resolution W4f core-level spectrum indicating W(VI) entities on the surface of the material. The percentage of P heteroatoms in **1** is small, and could not be detected in XPS. (It is observed, however, using other techniques, such as ESI-MS (see Supplementary fig. 12).

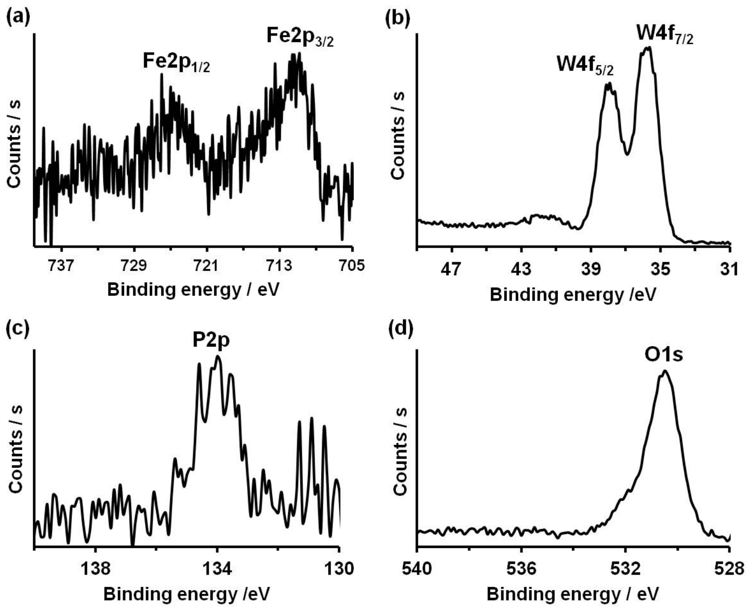

Supplementary Figure 18: High-resolution XPS spectra of $(TBA)_{10}[(PW_{11}O_{39}Fe)_2O]$ showing that **2** is comprised of Fe, W, P and O. (a) High-resolution Fe2p core-level spectrum indicates Fe(III) atoms. (b) High-resolution W4f core-level spectrum indicating W(VI) entities of iron-POM. (c) High resolution P2p spectrum indicating P(V) of the POM. (d) High-resolution O1s core-level spectrum. The O1s scan matches with a majority of O^{2-} ions in the POM, and few H_2O ligands possibly adsorbed as hydrated salt of iron-POM.

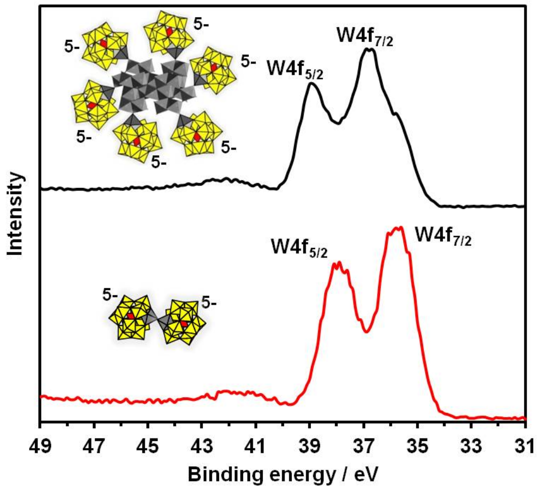

Supplementary Figure 19: Comparison of W4f scans of high-resolution XPS spectra of **1** (black curve; top) and $[(\alpha\text{-}PW_{11}O_{39}Fe)_2O]^{10-}$ (**2**) showing that both W4f scans are resembles to each other.

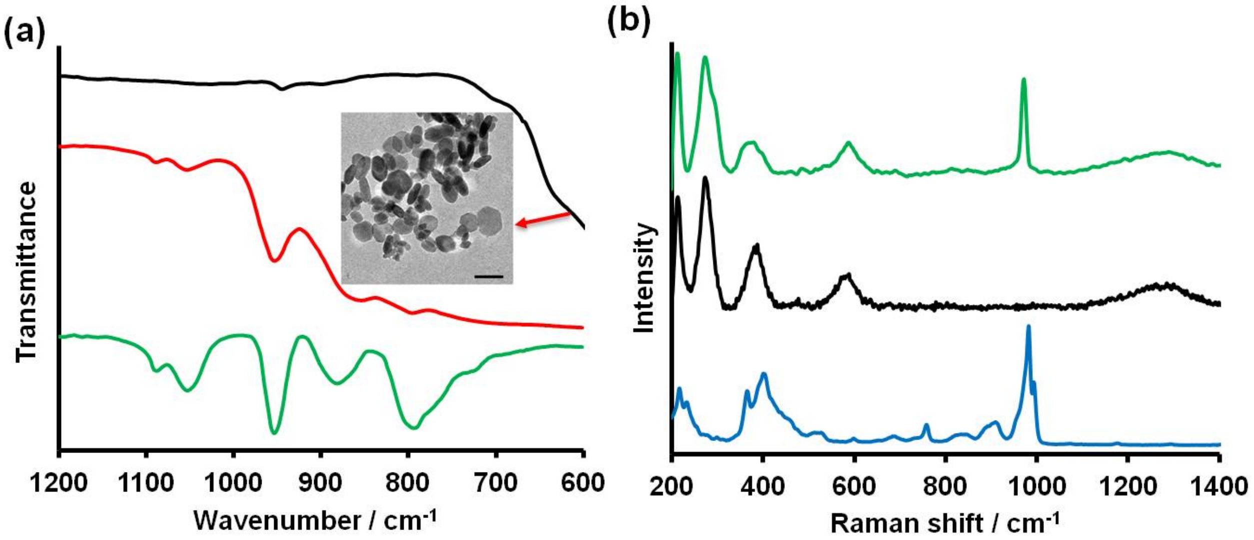

Supplementary Figure 20: Characterization of **1** by vibrational spectroscopy. (a) FTIR spectra of α-Fe_2O_3 (black curve), **1** unprocessed (red), and after baseline correction (green).

The FTIR spectrum of α-Fe_2O_3 whose (black curve) was obtained using α-Fe_2O_3 prepared under the same conditions (concentration, pH, temperature and time) as that used to prepare **1**, but without the presence of POM. The average particle size (see inset TEM image; scale bar: 100 nm) is much larger than the ca. 1.9 nm radius hematite cores of **1**. In the unprocessed (i.e., non-baseline corrected) FTIR spectrum of **1** (red curve), absorbance from the small iron-oxide NC cores is most pronounced starting from ca. 900 cm^{-1} and increases to a maximum (smaller %-transmittance) at ca. 750 cm^{-1}.

These two results (black and red curves) are consistent with FTIR spectra of α-Fe_2O_3 samples obtained using different preparative methods (and particle sizes), which are reported to give abrupt increases in absorbance (and smooth drops in transmittance) starting in some cases from 850 cm^{-1} (similar to **1**), and from 700 cm^{-1} in others (similar to our independently prepared α-Fe_2O_3; black curve). The shift in onset of absorbance to larger wavenumber values for **1**, relative to that of pure α-Fe_2O_3 (i.e., from 700 to ca. 900 cm^{-1}) could be due to small size of the hematite cores in **1**. The cores of **1** are comprised of ca. 300 Fe atoms, 25% of which (i.e., ca. 75 Fe atoms) are estimated to lie at the particle surface. Of those, ca. 15 are bound to POM ligands. The large percentage of Fe-O-Fe bonds of the cores at the particle surface would be expected to give rise to bands differing in energy from those within bulk α-Fe_2O_3.

Baseline correction was used to obtain the green curve, in which the relatively weaker intensity bands arising from the POM ligands are more clearly resolved. This baseline-corrected spectrum is presented in Fig. 3 of the text.

(b) Raman spectra of **1** (green), α-Fe_2O_3 (black), and $[(PW_{11}O_{39}Fe)_2O]^{10-}$ (blue). Comparison of the Raman spectra of **1** with that of α-Fe_2O_3 and of the molecular POM reveals the W=O stretching band of POM (sharp peak near 945 cm^{-1}) along with standard Raman-allowed stretches of α-Fe_2O_3 (200-600 cm^{-1}). The presence of one W=O stretching band in **1** instead of two—generally assigned to symmetric and anti-symmetric W=O stretches of molecular $[(PW_{11}O_{39}Fe)_2O]^{10-}$—indicates that the Raman-allowed modes for the POM bound to the hematite surface are different than for the molecular dimer.

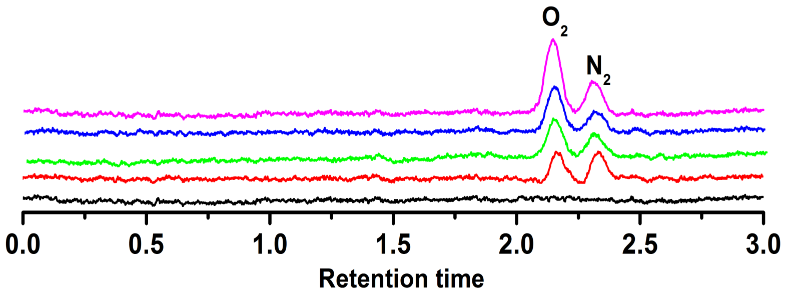

Supplementary Figure 21: Real-time measurement of photochemically produced O_2(g) by GC (thermo-conductivity detector, with Ar carrier gas). The black curve is a chromatogram of the headspace gas present after purging a solution of **1** in presence of 20 mM IO_4^- (pH 8) with argon (Ar) for 30 mins. The red curve is a chromatogram obtained after visible-light photo irradiation of the solution for 2 h. The green, blue and pink curves are chromatograms of gas samples injected after 4, 6 and 8 h of irradiation. In the chromatograms, residual N_2 is from air contamination during sampling (done by hand using gas-tight syringes). To handle this, quantification of the photochemically produced O_2 was achieved using a standard calibration curve prepared using different amounts of air (see Supplementary Fig. 19). The amount of O_2 associated with contamination by air was calculated from those calibration curves, and subtracted to obtain reported values of photochemically evolved O_2.

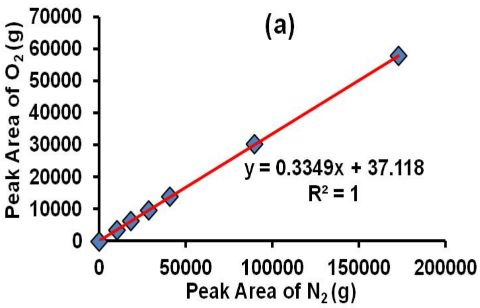

Supplementary Figure 22: Calibration curves used to quantify the amount of O_2 produced during photochemical reactions. Different amounts of air (20, 40, 60, 80, 150, and 300 μL) were injected using a gas tight syringe, and the peak areas for O_2 and N_2 were recorded. (a) Plot of O_2 peak area versus N_2 peak area. Linear fit of the data set (with R^2 = 1) gave a linear equation. That equation was then used to calculate the peak area of O_2 from air contamination during sampling of headspace gases from photochemical reactions. The area of O_2 from air was then subtracted from the O_2 peak areas observed during O_2-evolution reactions. (b) Plot of micro-moles of O_2 (g) versus peak area (assuming the composition of O_2 vs N_2 in ambient air is ca. 20.946 : 78.084) for different amounts of air injected into GC. Fitting of the data gave a straight line that was used to quantify the amount of photochemically produced O_2.

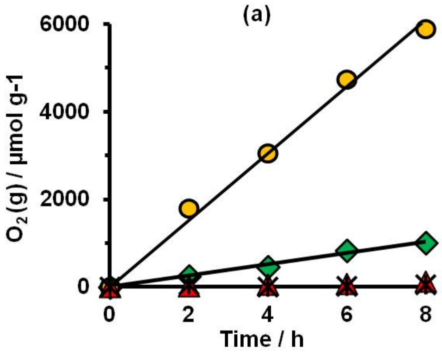

Supplementary Figure 23: Photochemically evolved O_2 (in µmol) as a function of time with **1** at pH 8 in presence of 20 mM periodate (yellow circles), 20 mM persulfate (green diamond), , and with pure $[\alpha\text{-}PW_{11}O_{39}Fe^{III}(H_2O)]^{4-}$ (Na^+ salt) and 20 mM periodate (red triangles with black stars).

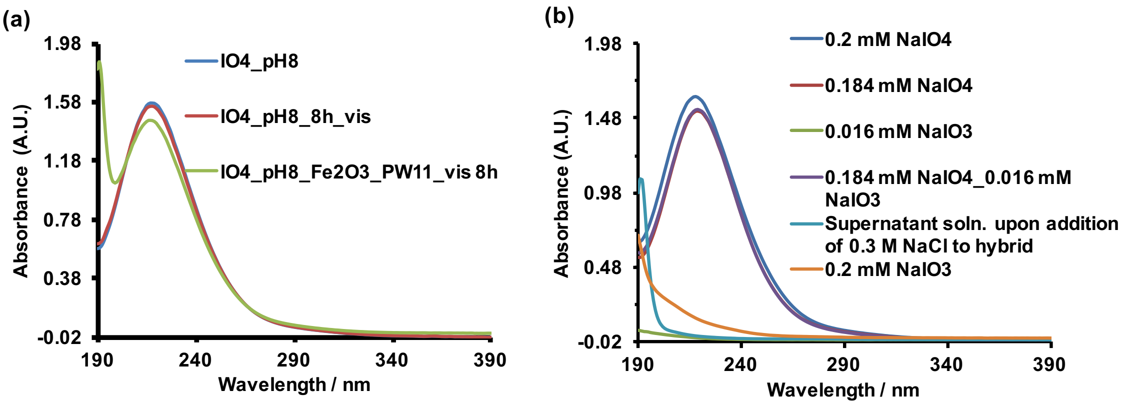

Supplementary Figure 24: Quantification of sodium periodate after photocatalytic water oxidation by **1**. (a) Blue curve: 0.2 mM $NaIO_4$ at pH 8 with an absorbance maximum (λ_{max}) at 218 nm. Red curve: 0.2 mM $NaIO_4$ after irradiation by visible-light for 8 h (a negligible change in absorbance indicates no significant loss of $NaIO_4$). Thereby, it demonstrates the photo-stability of $NaIO_4$ under our experimental conditions. Green curve: the periodate solution (100 times diluted to compare with the 0.2 mM standard solution) after photoreaction for 8h with 5.8 µM **1** and 20 mM $NaIO_4$ at pH 8. The un-reacted periodate was quantified by UV-vis spectroscopy after selective precipitation of **1** by NaCl. (The periodate absorbance at 218 nm after the photocatalytic reaction shows that 18.4 mM $NaIO_4$ remained unreacted. The amount reacted was then calculated to be two equivalents.) Control experiments: (b) UV-vis spectra of 0.2 mM $NaIO_4$ (blue curve), 0.184 mM $NaIO_4$ (red curve), 0.016 mM $NaIO_3$ (green curve), a mixture of 0.184 mM $NaIO_4$ and 0.016 mM $NaIO_3$ (purple curve), the supernatant solution (diluted 100x with water) obtained after selective precipitation of **1** (cyan curve), and 0.2 mM $NaIO_3$ (orange curve).

Supplementary Table 2: Calculation of quantum yield (Φ) with **1** and 5 nm α-Fe_2O_3[6] Using a 300 W Xe lamp with a 450 nm band pass filter.

	**1**	α-Fe_2O_3
Intensity of incident light (W cm^{-2})	0.00255	0.00255

Intensity of transmitted light (W cm^{-2})	0.00005	0
Illumination area (cm^2)	1	1
Light Intensity absorbed (W)	0.0025	0.00255
Energy of a photon (J)	4.41E-19	
No of photon absorbed	5.65E+15	5.78E+15
Energy of the photons (einstein s^{-1})	9.41E-09	9.60E-09
O_2 produces (mol s^{-1})	9.21E-11	1.12E-10
**Quantum yield (Φ)**	**3.91E-02**	**4.67E-02**

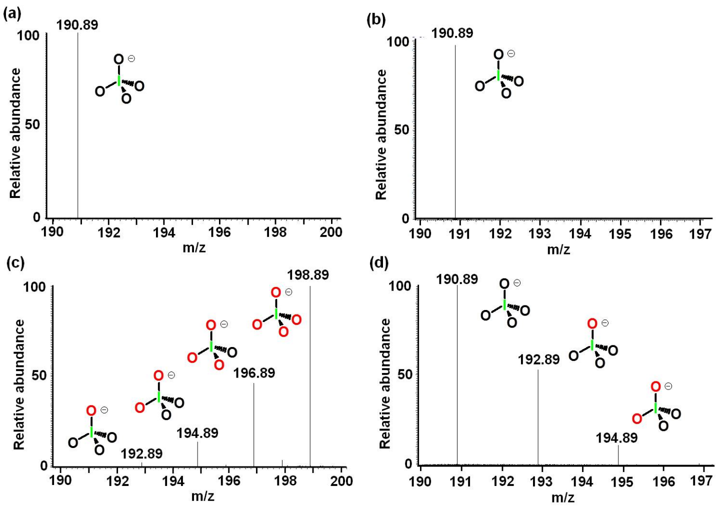

Supplementary Figure 25: ESI mass spectra (negative-ion mode) of IO_4^- in the presence of $H_2^{18}O$ and exchange of the oxide ligands of periodate with water 1 min after mixing (additional time-dependent data are reported in supplementary Table 3). (a) An acetonitrile solution of TBA periodate gave a predominant molecular ion peak at m/z = 190.89 corresponding to IO_4^- . (b) An aqueous (H_2O) solution of sodium periodate also gave a predominant molecular ion peak for IO_4^- . (c) The mass spectrum of a mixture of an acetonitrile solution of TBA periodate (0.2 μmoles) and 1.11 mmoles of $H_2^{18}O$. The dominant molecular-ion peak at m/z 198.89 indicates complete label exchange, giving $I^{18}O_4^-$ (red oxygen atoms represent ^{18}O), while presence of partially labeled periodate ions is consistent with the rapid exchange of oxygen atom of periodate with water. (d) ESI-mass spectrum of a mixture of an aqueous solution of sodium periodate (0.2 μmoles) and 0.6 mmoles of $H_2^{18}O$. The molecular ion peaks at m/z = 192.89 and 194.89 indicate the formation of $I^{18}OO_3$ and $I^{18}O_2O_2$ with a predominant molecular ion peak for IO_4^-. These data, obtained one min after additions of $H_2^{18}O$ to solution of periodate, confirm the rapid exchange of oxygen atom with water, even in acetonitrile.

Supplementary Table 3: Abundance and relative intensities of molecular ion peaks in ESI-mass spectra from combinations of periodate ion (IO_4^-) and $H_2^{18}O$ in acetonitrile and water[a] (see discussion in footnote "a").

Experiment		Abundance (relative intensity %) of ion peak at m/z				
	Time (min)	190.89; $[IO_4]^-$	192.89; $[I^{18}OO_3]^-$	194.89; $[I^{18}O_2O_2]^-$	196.89; $[I^{18}O_3O]^-$	198.89; $[I^{18}O_4]^-$
0.2 μmoles of $TBAIO_4$ in CH_3CN	-	2514442 (100)	-	-	-	-
Addition of 1.11 mmoles $H_2^{18}O$ into 0.2 μmoles of $TBAIO_4$ in CH_3CN	1	-	54506 (2)	240901 (11)	900785 (41)	2202566 (100)
	5	-	17963 (1)	158594 (7)	909080 (37)	2483884 (100)
	20	-	58996 (3)	305687 (14)	1009561 (45)	2180809 (100)
	120	-	17037 (1)	173682 (8)	911011 (42)	2192699 (100)
0.2 μmoles of $NaIO_4$ in H_2O	-	5554374 (100)	-	-	-	-
Addition of 0.6 mmoles $H_2^{18}O$ into 0.2 μmoles of $NaIO_4$ in H_2O	1	4536391 (100)	1823970 (40)	278246 (6)	-	-
	5	3676992 (100)	1899675 (52)	386088 (11)	-	-
	20	3298504 (100)	1744491 (53)	355780 (11)	-	-
	120	3494478 (100)	1789821 (51)	361005 (10)	-	-

[a]Addition of 1.11 mmoles of $H_2^{18}O$ to the solution of 0.2 μmoles TBA-IO_4 predominantly forms $I^{18}O_4^-$ within 1 min and the abundance and relative intensities of the peaks of $I^{18}OO_3^-$, $I^{18}O_2O_2^-$, $I^{18}O_3O^-$, and $I^{18}O_4^-$ remain unchanged with time indicating an equilibrium distribution resulting from rapid oxygen-exchange of periodate and water occurs in organic solvents, CH_3CN, as well as in water. As expected, oxygen atom exchange in pure water is rapid, and the relative intensities of the ions $I^{18}OO_3^-$, and $I^{18}O_2O_2^-$ remain unaltered after 5 mins, indicating that an equilibrium distribution is achieved rapidly. Use of a large excess of $H_2^{16}O$ with respect to $H_2^{18}O$ simply results in the presence of less $I^{18}OO_3^-$, and $I^{18}O_2O_2^-$ anions, and a more dominant IO_4^- signal. Note: Based on recent findings by Horvath (refs. 37 of the main text), orthoperiodate ($H_3IO_6^{2-}$) is the dominant species in water at pH 8. As such, the ESI-MS data reported here, in which only the IO_4^- species are observed, is an artifact of the method (either sampling or loss of water during ionization in the instrument). In any

event, rapid exchange in water (which is well known) is clearly observed, and in addition, we now show that exchange in acetonitrile is also rapid. Hence, that solvent can't be used to overcome the limitations that rapid exchange imposes on efforts to carry out definitive isotopic-labelling experiments.

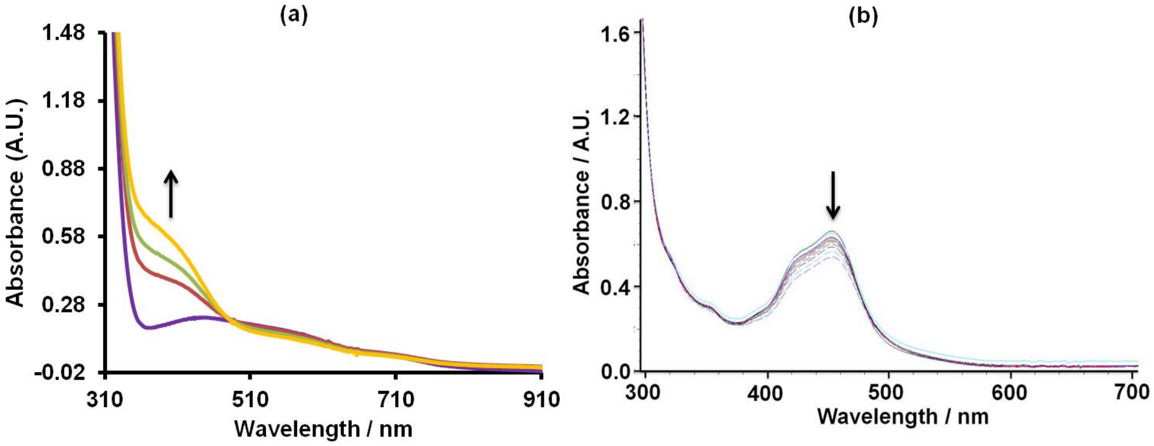

Supplementary Figure 26: (a) Chemical oxidation of [α-$AlV^{IV}W_{11}O_{40}]^{7-}$ to [α-$AlV^{V}W_{11}O_{40}]^{6-}$ by one-half equivalent $NaIO_4$. The purple solution of [α-$AlV^{IV}W_{11}O_{40}]^{7-}$ exhibits a broad absorbance over much of the visible range, with λ_{max} at 460 nm. Upon addition of $NaIO_4$ (to a final concentration 0.5 mM) to the purple solution of $[AlV^{IV}W_{11}O_{40}]^{7-}$ (final conc. = 1 mM), the solution slowly turns yellow, indicating the formation of $[AlV^{V}W_{11}O_{40}]^{6-}$. The reaction was carried out in a sealed quartz cuvette under purified Ar, and the headspace gas was checked by GC. No oxygen was produced during or after the reaction. (b) Visible-light driven oxidation of $[Ru^{II}(bpy)_3]Cl_2$ by $NaIO_4$. Reaction of 0.1 mM $[Ru^{II}(bpy)_3]Cl_2$ with 0.05 mM $NaIO_4$ was carried out using visible light irradiation (no reaction was observed in the dark). The decrease of the band at 452 nm (typical of $[Ru^{II}(bpy)_3]Cl_2$) was used to quantify the complete oxidation of two equivalents of Ru^{II} to Ru^{III}. In a separate experiment, using much more concentrated solutions of both reactants (10 and 20 mM, respectively), the photochemical reaction was performed in a sealed quartz cell in presence of purified Ar. After 2 h, no oxygen was observed, even though the number of electron equivalents transferred to IO_4^- (in principal) corresponded to ca. 0.5 μmol of O_2 (i.e., an easily detectable quantity). This shows that visible-light driven electron transfer to IO_4^- from a model photosensitizer (the Ru complex, as a model for the visible-light driven reduction of IO_4^- by hematite) occurs via a clean two-electron reduction, with no formation of O_2.

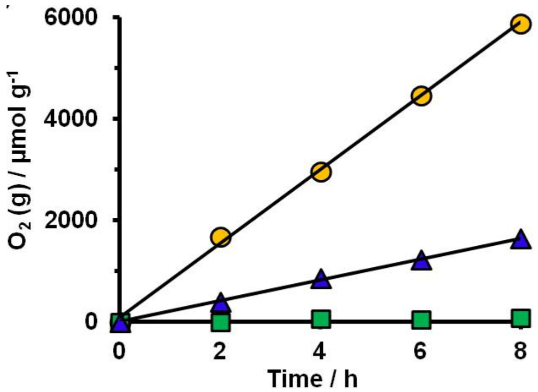

Supplementary Figure 27: Visible-light driven water oxidation in water, and in dry and wet MeCN. Yellow circles: O_2 evolved using the Na^+ salt of **1** in water at pH 8 in presence of 20 mM $NaIO_4$. Green squares: O_2 evolved using using the tetrahexylammonium salt of **1** in dry CH_3CN in presence of 20 mM $TBAIO_4$. No O_2 was produced. Blue triangle: O_2 evolved using the tetrahexylammonium salt of **1** in 1:1 v/v CH_3CN and H_2O, in presence of 20 mM $TBAIO_4$. Note: $TBAIO_4$ was prepared by reacting $NaIO_4$ with one equiv of $TBAHSO_4$.

Supplementary Table 4: Reported photochemical oxygen production rates for α-Fe_2O_3 NCs. **Note:** Some rates close to those reported below were obtained using a 300W Xe lamp. Our data (this work) were obtained using a 150W Xe lamp. For comparison and completeness, some rates reported for reactions carried out using UV light are also included. Reactions of hematite modified by other materials such as NiO or Au (as catalysts) are not included.

**Material**	**Particle size (nm)**	**Sacrificial oxidant**	**Light source & Power**	**Photochemical Oxygen production / Rate**	**Ref.**
i) [α-$PW_{11}O_{39}$Fe]-O^--α-Fe_2O_3 (**1**)<br>ii) Colloidal α-Fe_2O_3 [6]	i) 3 ± 0.5<br>ii) ca. 5.2	$NaIO_4$ (20 mM; pH 8)	150 W Xe lamp (λ > 420 nm) The illumination area is 1 cm^2 and the average light intensity is 566 mW cm^{-2}	i) 824.5 μmol g^{-1} h^{-1}<br>ii) 349.4 μmol g^{-1} h^{-1}	This work
i) α-Fe_2O_3 (Bulk-Fe_2O_3)<br>ii) α-Fe_2O_3 (Sonic-Fe_2O_3)<br>iii) α-Fe_2O_3 (Nano-Fe_2O_3)	i) 120<br>ii) 44<br>iii) 5.2	$AgNO_3$ (20 mM)	300 W Xe lamp (1.4 W/cm^2 at flask, λ 250–680 nm), measured with a UV/Vis GaAsP photodetector. Visible light (λ > 400 nm) via 400 nm long-pass filter	i) 250 μmol h^{-1} g^{-1} (λ ≥ 250 nm)<br>ii) 381 μmol h^{-1} g^{-1} (λ ≥ 250 nm)<br>iii) 1072 μmol h^{-1} g^{-1} (Note: λ ≥ 250 nm) ,and 767 μmol h^{-1} g^{-1} (λ > 400 nm)	10
α-Fe_2O_3 quantum dot (QD; oleic acid capping agent)<br>i) QD annealed at 800°C for 10 s<br>ii) QD annealed at 800C for 60 s<br>iii) QD annealed at 250C for 20 min<br>iv) Commercial α-Fe_2O_3	2 – 4	$AgNO_3$ (0.02 M)	300 W Xe lamp (**λ** > 420 nm, intensity 325 mW cm^{-2} illumination area 12.56 cm^2)	i) 310 μmol g^{-1} h^{-1}<br>ii) 150 μmol g^{-1} h^{-1}<br>iii) 190 μmol g^{-1} h^{-1}<br>iv) 130 μmol g^{-1} h^{-1}	11
α-Fe_2O_3 (capping agent: oleic acid / sodium acetate)<br>i) Nanocubes<br>ii) Nanoplates<br>iii) Nanoflakes<br>iv) Nanoparticles	i) (50 x 60 x 65)<br>ii) (200 x 200 x 20)<br>iii) (250 x 250 x 15)<br>iv) (35 x 40 x 50)	20.0 mM $AgNO_3$, [Cat.] = 10 mg	200 W Xe lamp (λ > 420 nm)	i) 37.5 μmol g^{-1} h^{-1}<br>ii) 10 μmol g^{-1} h^{-1}<br>iii) 9 μmol g^{-1} h^{-1}<br>iv) 42.5 μmol g^{-1} h^{-1}	12
i) α-Fe_2O_3<br>ii) Reduced grapheneoxide (rGO)/α-Fe_2O_3	i) 63.8 nm<br>ii) 41.4 nm (α-Fe_2O_3 particle on GO)	$AgNO_3$	300 W Xe lamp solar simulator (AM 1.5G filter), intensity 80 mW/cm^2	30.96 μmol g^{-1} h^{-1}<br>60.16 μmol g^{-1} h^{-1}	13
α-Fe_2O_3<br>(other rates reported in this article are faster, but are catalysed by Au)	25 nm α-Fe_2O_3	$Na_2S_2O_8$ (5.33 mM)	300 W Xe lamp (MAX-302, Asahi Spectra, USA) coupled with a UV cut-off filter (λ > 420 nm)	1030 μmol g^{-1} h^{-1}<br>(Note: lamp power is 2x that used in the present work.)	14
α-Fe_2O_3 in montmorillonite (MT, clay)	i) 30	Ag_2SO_4 (30 mM, pH =	150 W halogen lamp (light intensity, 357 mW cm^{-2})		15

i) 3 wt% amorph- α-Fe_2O_3/MT (calcination)<br><br>ii) 3 wt% α-Fe_2O_3/MT (hydrothermal)<br><br>iii) 3 wt% amorph- α-Fe_2O_3<br><br>iv) 25 wt% α-Fe_2O_3 (calcination)<br><br>v) 25 wt% α -Fe_2O_3 (hydrothermal)	ii) 60<br><br>iii) 300<br><br>iv) 30<br><br>v) 60	6.2)		i) 660 μmol g^{-1} h^{-1}<br><br>ii) 706 μmol g^{-1} h^{-1}<br><br>iii) 733 μmol g^{-1} h^{-1}<br><br>iv) 23.5 μmol g^{-1} h^{-1}<br><br>v) 32.2 μmol g^{-1} h^{-1}	
β-FeO(OH)	5-15	$NaIO_4$ (30 mM, pH 13)	300W Xe lamp (λ >400 nm 680 $mWcm^{-2}$)	1212 μmol g^{-1} h^{-1}	16

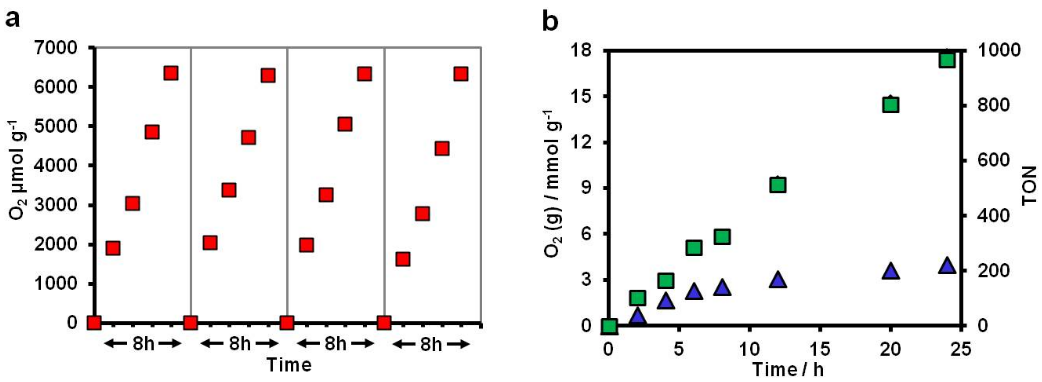

Supplementary Figure 28: (a) Dioxygen formed as a function of time during four successive reactions using the same sample of **1** (2.4 μM) quantitatively isolated and re-dissolved before each subsequent reaction (in 20 mM NaIO4). (b) Visible-light driven water-oxidation catalysis by **1** in compare to 5-nm colloidal α-Fe_2O_3[7]. Dioxygen produced per g of α-Fe_2O_3 in **1** (5.8 μM) during 24 h of reaction with 20 mM IO_4^- at pH 8 (green squares), and per g of 5-nm colloidal α-Fe_2O_3 (blue triangles).

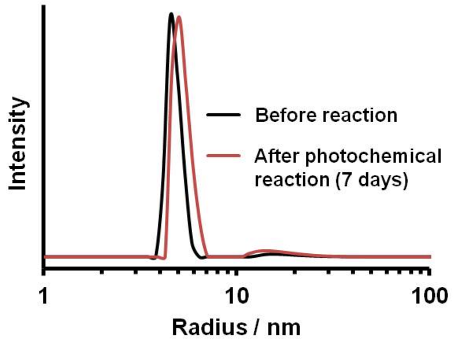

Supplementary Figure 29: DLS of an aqueous solution of **1** (black) before and (red) after photochemical reaction for 7 days. In both cases, the average number-weighted radius was. 4.9 ± 0.5 nm. (The initial size, before reactions, suggests that some aggregation may have occurred during multiple NaCl-precipitation / re-dissolution cycles prior to use of the material in catalysis. This is observed in some syntheses. Importantly, precipitation, or even cloudy solutions, has never been observed.)

Supplementary References

1 Haraguchi, N., Okaue, Y., Isobe, T. & Matsuda, Y. Stabilization of tetravalent cerium upon coordination of unsaturated heteropolytungstate anions. *Inorg. Chem.* **33,** 1015-1020, (1994).

2 Zonnevijlle, F., Tourne, C. M. & Tourne, G. F. Preparation and characterization of iron(III)- and rhodium(III)-containing heteropolytungstates. Identification of novel oxo-bridged iron(III) dimers. *Inorg. Chem.* **21,** 2751-2757, (1982).

3 Santos, F. M., Brandao, P., Felix, V., Domingues, M. R. M., Amaral, J. S. *et al.* Organic-inorganic hybrid materials based on iron(III)-polyoxotungstates and 1-butyl-3-methylimidazolium cations. *Dalton Trans.* **41,** 12145-12155, (2012).

4 Pichon, C., Dolbecq, A., Mialane, P., Marrot, J., Rivière, E. *et al.* Fe_2 and Fe_4 Clusters encapsulated in vacant polyoxotungstates: Hydrothermal synthesis, magnetic and electrochemical properties, and DFT calculations. *Chem. Eur. J.* **14,** 3189-3199, (2008).

5 Gamelas, J. A., Couto, F. A. S., Trovão, M. C. N., Cavaleiro, A. M. V., Cavaleiro, J. A. S. *et al.* Investigation of the thermal decomposition of some metal-substituted Keggin tungstophosphates. *Thermochim. Acta* **326,** 165-173, (1999).

6 Weinstock, I. A., Cowan, J. J., Barbuzzi, E. M. G., Zeng, H. & Hill, C. L. Equilibria between α and β isomers of Keggin heteropolytungstates. *J. Am. Chem. Soc.* **121,** 4608-4617, (1999).

7 Raming, T. P., Winnubst, A. J. A., van Kats, C. M. & Philipse, A. P. The synthesis and magnetic properties of nanosized hematite (α-Fe_2O_3) particles. *J. Colloid Interface Sci.* **249,** 346-350, (2002).

8 Raula, M., Gan-Or, G., Saganovich, M., Zeiri, O., Wang, Y. *et al.* Polyoxometalate complexes of anatase-titanium dioxide cores in water. *Angew. Chem. Int. Ed.* **54,** 12416-12421, (2015).

9 Pizzolato, E., Natali, M., Posocco, B., Montellano Lopez, A., Bazzan, I. *et al.* Light driven water oxidation by a single site cobalt salophen catalyst. *Chem. Commun.* **49,** 9941-9943, (2013).

10 Townsend, T. K., Sabio, E. M., Browning, N. D. & Osterloh, F. E. Photocatalytic water oxidation with suspended α-Fe_2O_3 particles-effects of nanoscaling. *Energy Environ. Sci.* **4,** 4270-4275, (2011).

11 Wang, J., Zhang, N., Su, J. & Guo, L. α-Fe_2O_3 quantum dots: low-cost synthesis and photocatalytic oxygen evolution capabilities. *RSC Adv.* **6,** 41060-41066, (2016).

12 Xiang, Q., Chen, G. & Lau, T.-C. Effects of morphology and exposed facets of α-Fe_2O_3 nanocrystals on photocatalytic water oxidation. *RSC Adv.* **5,** 52210-52216, (2015).

13 Meng, F., Li, J., Cushing, S. K., Bright, J., Zhi, M. *et al.* Photocatalytic water oxidation by hematite/reduced graphene oxide composites. *ACS Catal.* **3,** 746-751, (2013).

14 Cao, S.-W., Fang, J., Shahjamali, M. M., Wang, Z., Yin, Z. *et al.* In situ growth of Au nanoparticles on Fe_2O_3 nanocrystals for catalytic applications. *CrystEngComm* **14,** 7229-7235, (2012).

15 Kakuta, S. & Abe, T. Photocatalysis for water oxidation by Fe_2O_3 nanoparticles embedded in clay compound: correlation between its polymorphs and their photocatalytic activities. *J. Mater. Sci.* **44,** 2890-2898, (2009).

16 Shelton, T. L., Bensema, B. L., Brune, N. K., Wong, C., Yeh, M. *et al.* Photocatalytic water oxidation with iron oxide hydroxide (rust) nanoparticles. *J. Photonics Energy* **7,** 012003, (2016).
